# Friction‐Assisted Liquid Metal‐Driven Anchoring of Low Redox Potential Metal Ions for Enhanced Electromagnetic Wave Absorption

**DOI:** 10.1002/advs.202511810

**Published:** 2025-08-11

**Authors:** Tao Zhang, Geng Chen, Lechun Deng, Limin Zhang, Hao Shen, Qiang Chen, Hu Liu, Hongjing Wu

**Affiliations:** ^1^ Key Laboratory of Green and High‐end Utilization of Salt Lake Resources Qinghai Engineering and Technology Research Center of Comprehensive Utilization of Salt Lake Resources Qinghai Institute of Salt Lakes Chinese Academy of Sciences Xining 810008 China; ^2^ MOE Key Laboratory of Material Physics and Chemistry under Extraordinary Conditions School of Physical Science and Technology Northwestern Polytechnical University Xi'an 710072 China; ^3^ State Key Laboratory of Solidification Processing School of Materials Science and Engineering Northwestern Polytechnical University Xi'an 710072 China; ^4^ Department of Applied Physics School of Science Chang'an University Xi'an 710064 China

**Keywords:** electromagnetic wave absorption, friction‐assisted strategy, liquid metal, low redox potential, polarization loss

## Abstract

Overcoming the fundamental thermodynamic‐kinetic dilemma restricting metal ion reduction/anchoring (MIRA) strategies is critical for advancing next‐generation technologies reliant on precise electron transfer and stable interfaces. However, the persistent challenge in conventional approaches lies in the concurrent inhibition of thermodynamically reactions, occurrence of undesired kinetic pathways, and compromised anchoring efficiency. Here, a paradigm of friction‐assisted utilizing gallium‐indium liquid metal (LM) to circumvent these constraints, enabling efficient MIRA of low reduction potential (LRP) ions. This study elucidates the mechanism by which friction‐assisted LM promotes the MIRA of LRP, overcoming thermodynamic barriers, suppressing parasitic reactions, and enabling efficient anchoring. Building on this principle, InGaZn_5_O_8_ with a distinct crystalline structure is synthesized, whose unique electronic configuration engenders enhanced electromagnetic wave absorption. A concentration‐dependent dual effective absorption bandwidth (EAB) phenomenon is observed, and optimized LM‐Zn‐8 achieves an EAB of 5.92 GHz at a minimal thickness of 1.3 mm and a minimum reflection loss (RL_min_) of ‐44.44 dB. Furthermore, the friction‐assisted strategy demonstrates broad applicability to diverse LRP ions (e.g., Al^3^⁺, Cr^3^⁺), establishing a universal and customizable platform for fabricating MIRA composites with tailored functionalities across a wide range of applications.

## Graphical abstract

Friction‐assisted liquid metal resolves the thermodynamic‐kinetic dilemma of low reduction potential (LRP) ions through shear‐induced electron generation, enabling efficient LRP reduction/anchoring while suppressing parasitic reactions. This approach synthesizes InGaZn_5_O_8_ crystals with unique electronic configurations, achieving a 5.92 GHz effective absorption bandwidth at 1.3 mm. Applicable to Al^3+^ and Cr^3+^, it provides a versatile platform for functional microwave absorbing composites.

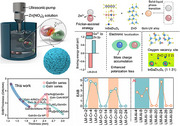

## 1 Introduction

The precise manipulation of the electron transfer process and optimization of interface stability lies at the heart of advancing next‐generation applications in catalysis, energy storage, corrosion protection, electromagnetic (EM) wave absorption, and others.^1^, ^2^, ^3^ ^]^ The metal ion reduction/anchoring (MIRA) strategy fundamentally enhances material functionalities by enabling electronic structure optimization, reinforcing interfacial stability, and triggering microstructural reconstruction‐crucially addressing the core demands outlined above.^[^ ^4^, ^5^ ^]^ However, the current implementation of MIRA faces a trilemma of thermodynamic‐kinetic coordination. Significant thermodynamic barriers arising from redox potential mismatches between metal ions and substrates hinder reaction progression, while the limited distribution of defect/edge sites suppresses anchoring efficiency, constraining full utilization of active sites. Although external energy input can overcome these barriers, it often activates non‐selective pathways, generating undesired byproducts and compromising interfacial stability, which collectively pose kinetic instability risks and impede practical implementation.^[^ ^6^, ^7^, ^8^, ^9^, ^10^ ^]^ Addressing these challenges necessitates the development of innovative paradigms to synergistically lower thermodynamic barriers, particularly for low reduction potential (LRP) ions, while optimizing kinetics to suppress side reactions and enhance anchoring efficiency.

Leveraging dynamic charge transfer at frictional interfaces, the friction‐assisted strategy emerges as a novel charge regulation approach, which is expected to overcome the fundamental trilemma and advance MIRA implementations, particularly for challenging LRP systems. Resolving the MIRA trilemma demands rigorous theoretical and methodological considerations of the friction‐assisted strategy's feasibility. i) Interfacial friction serves as an energy source where localized heating and electron excitation generate substantial free electrons, significantly lowering activation barriers for LRP ion reduction to overcome thermodynamic constraints; ii) Differential charge confinement capacities between friction‐components enable directional charge migration under highly localized/transient energy input, allowing precise metal ion capture while suppressing side reactions for thermodynamic stabilization; iii) During friction, shear‐induced continuous surface renewal maximizes utilization of anchoring sites through dynamically active interfaces, thereby optimizing MIRA efficacy. Guided by these theoretical insights, the friction‐assisted strategy overcomes thermodynamic constraints through mechanical to chemical energy conversion while establishing directional ion transport pathways via interfacial charge confinement gradients, thereby resolving kinetic limitations and enabling precise MIRA.

Friction sensitive gallium‐indium liquid metal (LM), exhibiting inherent electron gas behavior, serves as an ideal platform for implementing the aforementioned strategy.^[^ ^11^, ^12^ ^]^ Interfacial friction between gallium and indium inducing charge separation, generating transient and highly localized electric fields.^[^ ^13^, ^14^, ^15^ ^]^ This high‐energy state accumulated at frictional interfaces is captured by metal ions, thus reducing their reduction barrier. Meanwhile, the electron saturation state inherent to Ga/In creates an energetic funnel that selectively directs electrons toward LRP ions, enabling precise charge transfer while suppresses parasitic side reactions that plague conventional approaches. Subsequently, LM at interfacial friction will triggers spontaneous solid‐liquid phase transitions, post‐phase transition, the dynamic interfacial of GaIn LM provides abundant active sites that through metal‐coordination interactions, facilitate both electrostatic attraction and adequately stable anchoring of LRP at the LM interface. By exploiting LM's unique friction properties, this strategy establishes well‐defined electron transport channels, which effectively overcome the thermodynamic reduction potential barrier, suppress parasitic reactions, and achieve a large amount of metal ion capture.

In this research, we present a friction‐assisted LM strategy for the MIRA of LRP, systematically exploring their potential in EM wave absorption. Capitalizing on LM's mechanosensitive properties, we engineered a friction‐induced reduction and anchoring mechanism that harnesses transient high‐local electric fields and abundant free electrons generated though interfacial friction. This approach overcomes inherent thermodynamic barriers in conventional systems, enabling direct Zn^2^⁺ incorporation into the LM lattice while eliminating parasitic side reactions. Benefiting from abundant active sites at dynamic interfaces, Zn^2+^ is effectively anchored on the LM surface, forming a distinct InGaZn_5_O_8_ phase. This distinct structure manifests experimentally in binding energy shifts, high concentration oxygen vacancy, and lattice strain/stress demonstrate Zn/Ga substitution induced charge symmetry breaking and defect‐induced relaxation for boosting EM wave dissipation. Theoretical verification confirms atomic scale charge localization at Zn sites and work function mismatch driven interfacial charge redistribution, enhancing polarization mechanisms. Therefore, the optimized LM‐Zn‐8 achieves an exceptionally effective absorption bandwidth (EAB) of 5.92 GHz at a minimal thickness of 1.3 mm and a minimum reflection loss (RL_min_) of −44.44 dB. With an thickness‐normalized EAB of 4.55 GHz mm^−1^, it significantly surpasses existing GaIn and GaInSn LM absorbers. Moreover, this strategy exhibits robust universality by effectively addressing high valence/LRP ions (e.g., Cr^3+^, Al^3+^) beyond the scope of conventional MIRA systems, while maintaining concentration dependent dual EAB bands across all tested configurations, thereby demonstrating scalable performance. This work not only establishes a novel friction‐assisted approach for MIRA of LRP in LM systems but also elucidates the underlying mechanisms of friction‐assisted MIRA, offering a reasonable explanation for its application in the field of EMW absorption.

## 2 Results and Discussion

### 2.1 Friction‐Assisted LM Enables the Reduction and Anchoring of LRP Metal Ions

**Figure 1:**
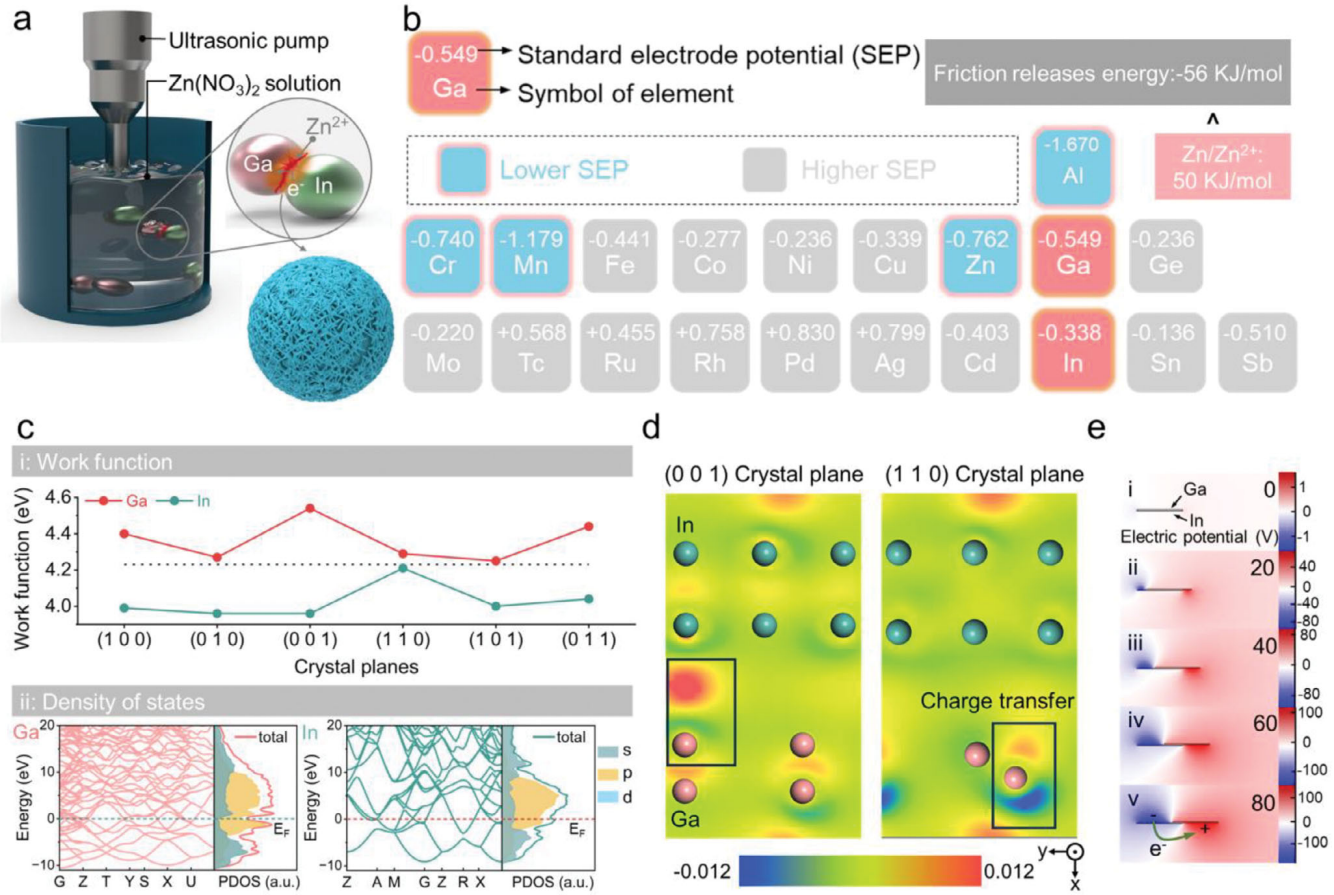
a) Schematic illustration of the synthesis process for LM‐X‐y; b) Standard electrode potentials of selected elements: red for Ga/In, blue for low redox potential metal ions, and gray for high redox potential metal ions; c) Work function of Ga/In across different crystal planes and corresponding surface state densities; d) Differential charge density distributions of Ga/In on the (001) and (110) crystal planes; e) Electric potential distributions for different friction distance, simulated using COMSOL Multiphysics software.

In an ultrasonic apparatus, the MIRA of LRP using friction‐assisted LM was achieved, as illustrated in **Figure** **1**a. Solid Ga/In particles, serving as friction components dispersed in solutions containing target metal ions at varying concentrations, undergo interparticle friction driven synergistically by ultrasound‐induced cavitation effects, localized polar pressure, and oscillatory shear forces. This process facilitates their solid‐liquid phase transition, ultimately forming a liquid GaIn alloy. Based on Gibbs free energy calculations, Zn^2^⁺ was selected as a model ion due to its low valence state and small standard electrode potential (SEP) difference with Ga/In (Figure 1b).^[^ ^16^ ^]^ The friction‐generated energy (−56 kJ mol^−1^) exceeds the energy required for Zn^2^⁺ reduction (50 kJ mol^−1^), confirming the feasibility of friction‐assisted reduction and anchoring.^[^ ^17^ ^]^ This strategy was further validated for other LRP metals (Cr^3^⁺, Al^3^⁺, and Mn^2^⁺) via extended experiments, establishing a universal approach for realizing MIRA of LRP. Moreover, to validate that friction induced high‐energy transient localized electric fields generate abundant free electrons, facilitating Ga/In solid‐liquid phase transitions and providing the critical energy input for low reduction potential metal ion reduction and anchoring, a series of comparative experiments were designed (Figure S1, Supporting Information).

Additionally, the feasibility of Ga/In as a friction component for realizing the MIRA of LRP was confirmed as follow. As illustrated in Figures 1c and S2 (Supporting Information), density functional theory (DFT) calculations reveal that Ga exhibits a higher work function (WF) than In across all crystal planes, establishing a thermodynamically favorable electron transfer pathway from In to Ga, which is the prerequisite for Zn^2^⁺ reduction. Meanwhile, the density of states (DOS) exhibits typical metallic conductivity, with the Fermi level crossing the projected density of states (PDOS) for efficient charge exchange, and high PDOS intensity implies strong electronic coupling.^[^ ^18^, ^19^ ^]^ To investigate the selectivity of electron transport, differential charge density maps clearly show the electron migration behavior along the transport path from In to Ga. (Figures 1d and S3, Supporting Information). Macroscopic COMSOL finite element simulations demonstrate that electrostatic induction generates positive potentials on Ga and negative potentials on In, driving electron transfer from low (In) to high (Ga) potential (Figures 1e and S4, Supporting Information).^[^ ^20^, ^21^ ^]^ Therefore, the In to Ga charge transfer creates an electron‐rich environment, where electrons accumulated on Ga surfaces preferentially reduce adsorbed Zn^2^⁺ to lower valence states. Meanwhile, electrostatic attraction between the friction interface and Zn^2^⁺ enhances ion migration to the interface, with frictional energy overcoming kinetic barriers for reduction and stabilization.^[^ ^20^, ^21^ ^]^ For clarity, the samples prepared form LRP metal ion with varying concentration are designated as LM‐X‐y, where X represents the metal ion and y denotes the concentration (Table S1, Supporting Information).

### 2.2 Structural and Compositional Characterization of Friction‐Assisted LM for LRP Ion Capture

**Figure 2:**
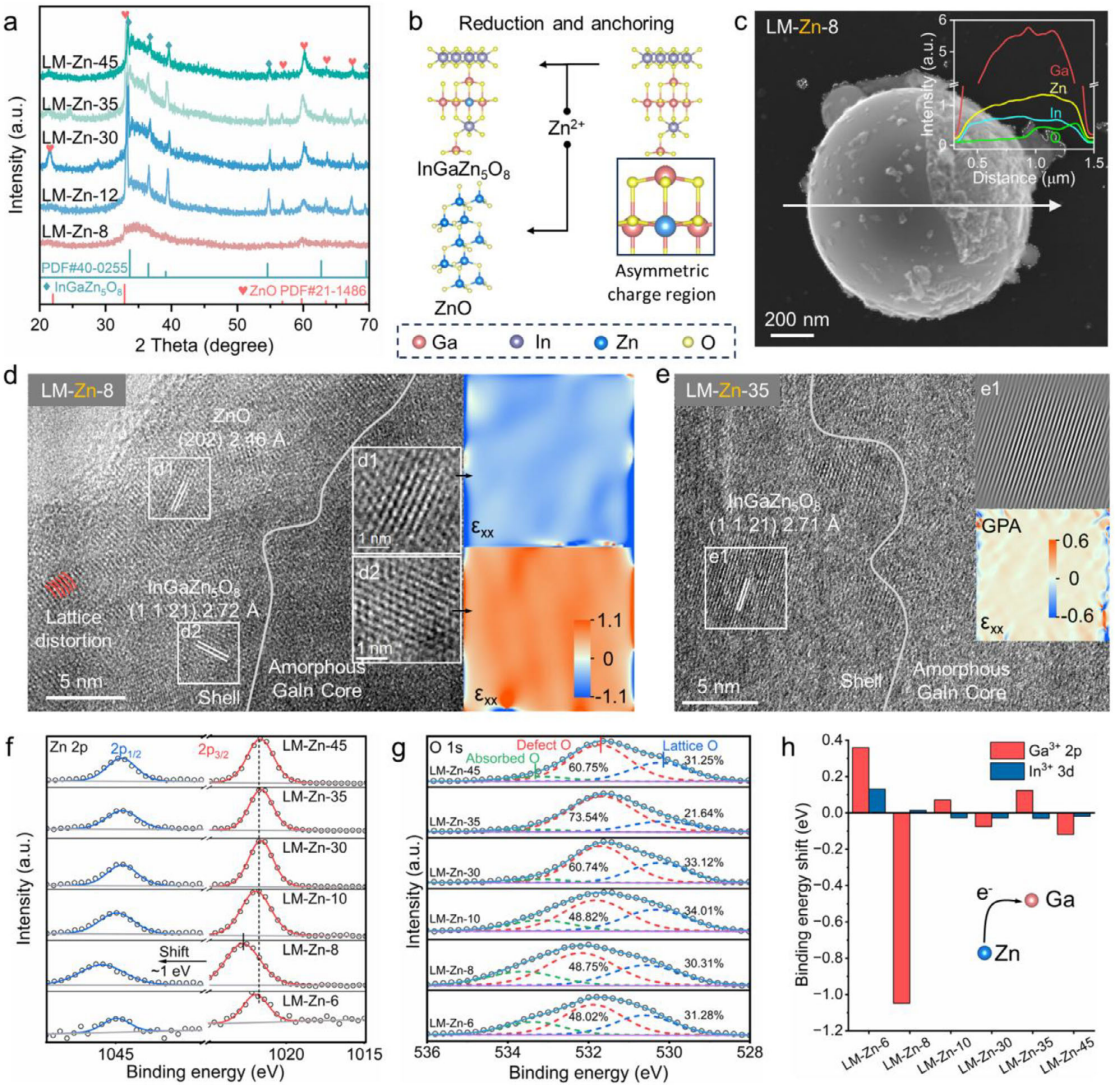
a) XRD patterns of samples synthesized via friction‐assisted LM reduction and Zn^2+^ anchoring; b) Schematic illustration of Zn salt reduction and ion anchoring mechanism; c) SEM image and line‐scan elemental profile of LM‐Zn‐8; d, e) High‐resolution TEM images and geometric phase analysis of LM‐Zn‐8 and LM‐Zn‐35; f) High‐resolution XPS spectra of Zn 2p for LM‐Zn; g) High‐resolution XPS spectra of O 1s showing absorbed oxygen, defect oxygen, and lattice oxygen in LM‐Zn; h) Comparative binding energy shifts of Ga^3+^ 2p and In^3+^ 3d.

The XRD patterns directly characterized the phase components of the reduced and anchoring Zn ion in the LM‐Zn system. As shown in **Figures** **2**a and S5 (Supporting Information), varying the concentrations of Zn salt doesn't change the products, which consisted of InGaZn_5_O₈ (PDF#40‐0255), ZnO (PDF#21‐1486), and an amorphous GaIn LM forming near 35°.^[^ ^22^, ^23^, ^24^ ^]^ Frictional energy induces the solid‐liquid phase transition of Ga/In, and based on their intrinsic oxidation propensity, the surface layer generates gallium oxide and indium oxide with lattice vacancies and interstitial defects. Since the reduced Zn atoms (1.34 Å) had a similar atomic radius to that of Ga (1.35 Å), they were anchored through lattice substitution/interstitial, formed stable metallic bonds with the LM, and yielded stable InGaZn_5_O₈. The substitutional and interstitial integration of structurally distinct atoms triggers crystallographic symmetry breaking, thereby generating localized asymmetric charge distributions (Figure 2c). Meanwhile, the local electric field during friction drove the reaction between Zn ions and dissolved oxygen to yield ZnO. The morphological structure of the LM‐Zn was analyzed by scanning electron microscopy (SEM), as depicted in Figures 2c and S6–S14 (Supporting Information). Under different Zn salt concentration treatments, the microstructures are highly similar, with a uniform irregular undulating, and granular structure formed on the surface. Line scanning technique was adopted to detect the energy intensity of surface elements. The results show that the content of Ga element is significantly higher than that of other elements, which is mainly due to the property that LM Ga is prone to surface oxidation. Elemental line scanning reveals that the surface enrichment zone exhibits elevated oxygen and zinc intensities, thereby indicating the irregular undulating particles in LM‐Zn primarily consist of Zn/O compounds. In addition, energy dispersive spectroscopy (EDS) mapping analysis further confirms that various elements are well dispersed in the material (Figure S6–S14, Supporting Information).

To clarify the phase composition of the friction‐assisted LM reduction and anchoring of Zn salt, transmission electron microscopy (TEM) analysis was performed. Combining high‐resolution TEM (HR‐TEM) and EDS mapping, we could inference that the outer shell of the friction‐assisted LM products is identified as InGaZn_5_O₈ and ZnO, exhibiting distinct (1 1 21) and (2 0 2) lattice fringes, while the core consists of amorphous GaIn LM (Figures 2d,e and S15–S19, Supporting Information). Furthermore, the LM‐Zn sample exhibits minimal lattice distortion, demonstrating high crystallographic integrity. Such low lattice strain facilitates enhanced intergranular carrier transport, thereby strengthening the material's EM response. Geometric phase analysis (GPA) analysis of HR‐TEM images (Figure 2d,e) demonstrates that ZnO and InGaZn_5_O_8_ lattices exhibit no significant strain variations along the ε_xx_ direction, even though Zn substitution of Ga, which is driven by their comparable atomic sizes, could potentially induce lattice distortion. The color of ZnO ranges from white to blue, while InGaZn_5_O₈ transitions from white to orange, corresponding to compressive and tensile strains, respectively, further verifying the strong carrier transport capability in the samples.^[^ ^25^ ^]^ Based on the previous analyses and the crystal structure formula of InGaZn_5_O₈ and ZnO, it has been effectively demonstrated that Zn salt is reduced and anchored within the crystalline phase via the friction‐assisted strategy.

To elucidate the chemical state evolution of Zn and its anchoring mechanism, X‐ray photoelectron spectroscopy (XPS) was employed to probe the valence states of surface elements of all samples. The high‐resolution XPS spectra exhibit distinct doublet spin‐orbit peaks at 1045.0 and 1022.0 eV, corresponding to the Zn 2p_1/2_ and Zn 2p_3/2_ (Figures 2f and S23, Supporting Information).^[^ ^26^ ^]^ The O 1s region reveals three distinct components: surface‐adsorbed oxygen (533.2 eV), defect‐associated oxygen (531.8 eV), and lattice oxygen (530.4 eV).^[^ ^27^ ^]^ Notably, the lattice oxygen content exhibits minimal variation (∼30%) with a consistent binding energy, indicating that Zn salt concentration variations have no significant impact on the fundamental oxygen coordination environment within the crystal structure (Figures 2g and S20, Supporting Information). Minor variations in the proportion of lattice oxygen might be associated with microscopic structural factors such as oxygen vacancies in the crystal structure. In contrast, the proportion of defect oxygen exhibited significant fluctuations, and these defect oxygen species can act as trapping centers, effectively capturing and accumulating carriers, thus enabling the efficient attenuation of EMW. The XPS spectra of Ga 2p and In 3d confirmed that Ga/In particles played a role in the friction‐assisted reduction and anchoring of Zn^2+^ by LM.^[^ ^28^ ^]^ There was evident charge migration among Ga/In particles, inducing a transition of Ga and In from the initial 0 valence state to +3 valence state, while retaining the intrinsic properties of the 0 valence LM (Figure S21, Supporting Information). A systematic statistical analysis of the binding energy shifts of Ga^3+^ and In^3+^ showed that most samples had no significant binding energy shifts, suggesting similar chemical environments (Figure S22, Supporting Information). Intriguingly, LM‐Zn‐8 exhibits a unique electronic perturbation: Zn 2p shifts upward by ≈1 eV, while Ga^3+^ 2p shifts downward by 1 eV. This anti‐correlated binding energy shift arises from Zn^2+^ substitution at Ga^3+^ lattice sites, the lower charge of Zn^2+^ reduces Coulombic repulsion with adjacent anions, strengthening local Zn─O interactions and compensating for the charge imbalance.^[^ ^29^ ^]^ Consequently, the increased electron density around Ga sites lowers its binding energy. Such targeted electronic modulation disrupts local charge symmetry in the material structure, enabling precise tailoring of polarization behavior and electron transport properties, thereby determining the material's EM response.

### 2.3 EMW Absorption Properties of the Prepared by Friction‐Assisted LM MIRA of LRP

**Figure 3:**
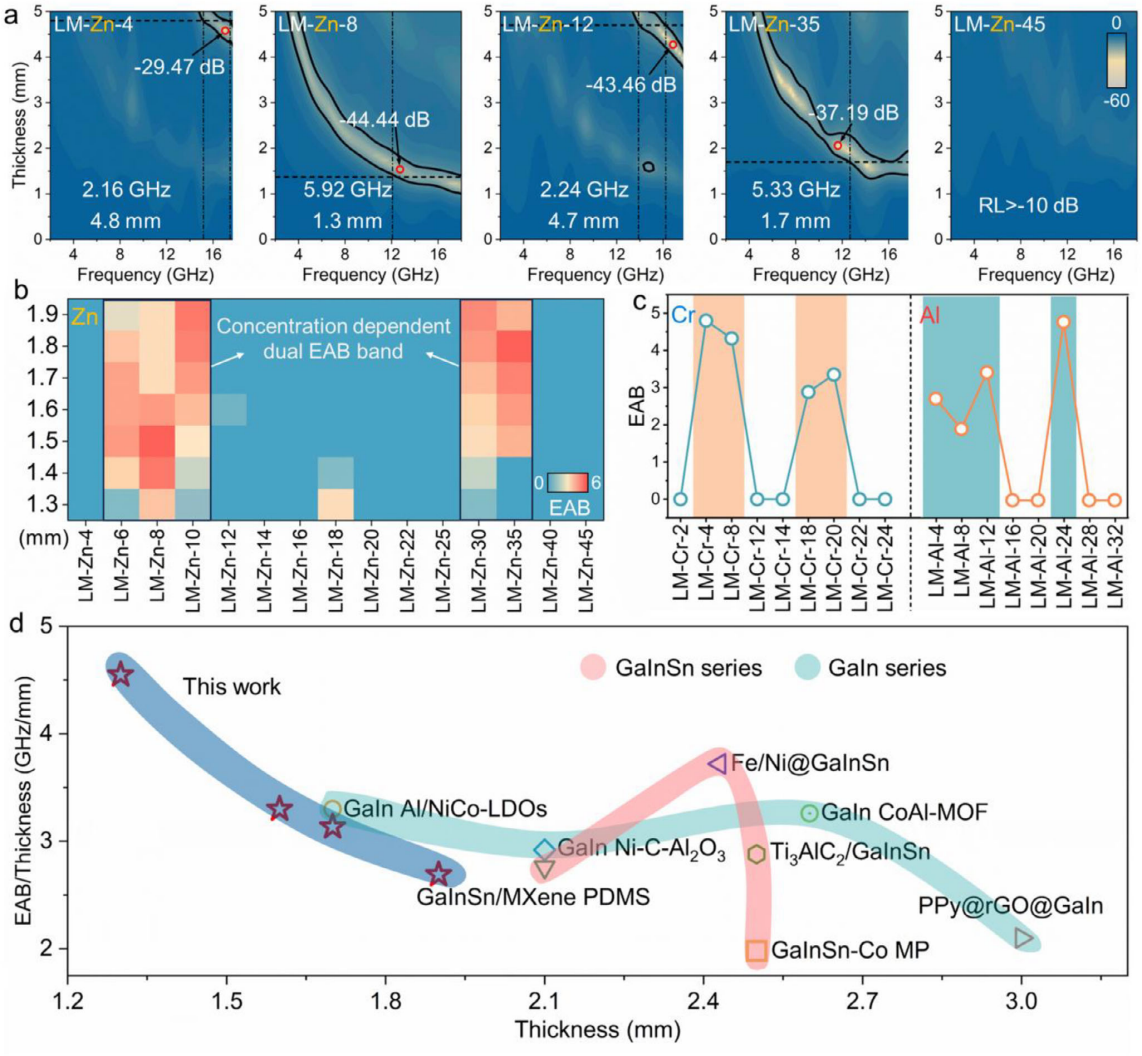
a) Reflection loss and EAB values of LM‐Zn‐6, LM‐Zn‐8, LM‐Zn‐10, and LM‐Zn‐35 calculated using transmission line theory.; b) Thermograms showing EAB values of the LM‐Zn system at different thicknesses; c) Performance variation patterns of the LM‐Cr and LM‐Al Systems; d) EAB/thickness and thickness comparison of the LM‐Zn system with other LM‐based EMW absorbing materials.

To comprehensively elucidate the reduction and anchoring effect of friction‐assisted LM on LRP metal ions (e.g., Zn^2^⁺, Cr^3+^, Al^3+^) and its impact on EMW absorption, we systematically evaluated the EMW absorption performance of all samples using transmission line theory.^[^ ^30^ ^]^ As depicted in **Figures** **3**a, S25 and S26 (Supporting Information), precise modulation of Zn salt concentration induced a distinctive “enhancement, weakening, re‐enhancement, degradation” non‐monotonic trend in EMW absorption. This phenomenon, consistently observed in both EAB and minimum reflection loss (RL_min_) across multiple thicknesses (Figures 3b and S27, Supporting Information), is defined as the “concentration dependent dual EAB band” effect. Specifically, under low Zn salt concentration, LM‐Zn‐4 demonstrates a limited EAB of 2.16 GHz at 4.8 mm thickness. Increasing Zn salt concentration promotes the formation of uniformly dispersed ZnO on GaIn LM surfaces (Figure S25, Supporting Information). These ZnO facilitate local charge transfer and enhance interfacial polarization between GaIn and InGaZn_5_O_8_/ZnO, yielding an EAB of 5.92 GHz with a RL_min_ of −44.46 dB at only 1.3 mm thickness. Further elevation of Zn salt concentration drives denser Zn species distribution within the GaIn LM matrix, accompanied by increased ZnO phase formation. This structural densification impedes interfacial electron transport, thereby reducing EM wave absorption. At higher concentrations, LM‐Zn particles transition to irregular spherical morphologies due to excessive Ga consumption in GaIn LM, disrupting structural homogeneity. Concurrently, outward oxide growth compromises surface oxide layer compactness, modulating interfacial electron transport dynamics. This reconfiguration enables LM‐Zn‐35 to achieve a 5.33 GHz EAB at 1.7 mm thickness. Finally, the overabundance of ZnO, characterized by inherently low dielectric permittivity, diminishes EM absorption performance.

For practical applicability assessment, radar cross section (RCS) simulations were performed to the signal echo intensity of the coatings.^[^ ^31^ ^]^ The results show that for the LM‐Zn‐8 under the condition of using a perfect electric conductor as the backplane, its echo scattering and absorption signal is significantly reduced (the minimum is −43.36 dB m^2^ at 76°), validating their feasibility for real‐world applications under far‐field conditions (Figure S28, Supporting Information). Meanwhile, to verify the university of the friction‐assisted LM approach for the MIRA of LRP, experimental validations were carried out on other metal ions based on Gibbs free energy calculations. By systematically adjusting the concentration, we successfully achieved the controllable synthesis of absorbers for the higher valent Cr^3+^ and Al^3+^ with lower SEP. The experimental results demonstrate that other metal ions with LRP also exhibit a characteristic concentration dependent dual EAB windows, which conclusively validates the feasibility and general applicability of the friction‐assisted strategy for MIRA of LRP. (Figures 3c, S29 and S30, Supporting Information). Building on these findings, the strategy of friction‐assisted LM for MIRA of LRP demonstrates remarkable potential in electromagnetic absorption. To comprehensively evaluate the EMW absorption performance, we conducted systematic comparisons with previously reported LM‐based and other absorbers.^[^ ^17^, ^32^, ^33^, ^34^, ^35^, ^36^, ^37^, ^38^, ^39^, ^40^, ^41^, ^42^, ^43^, ^44^, ^45^, ^46^, ^47^ ^]^ The results reveal that this approach endows the materials with competitive advantages in both absorption bandwidth and ultrathin thickness (Figures 3d, S32, Tables S3, and S4, Supporting Information).

### 2.4 Electromagnetic Response Modulation via Friction‐Assisted LM Anchoring Strategy

**Figure 4:**
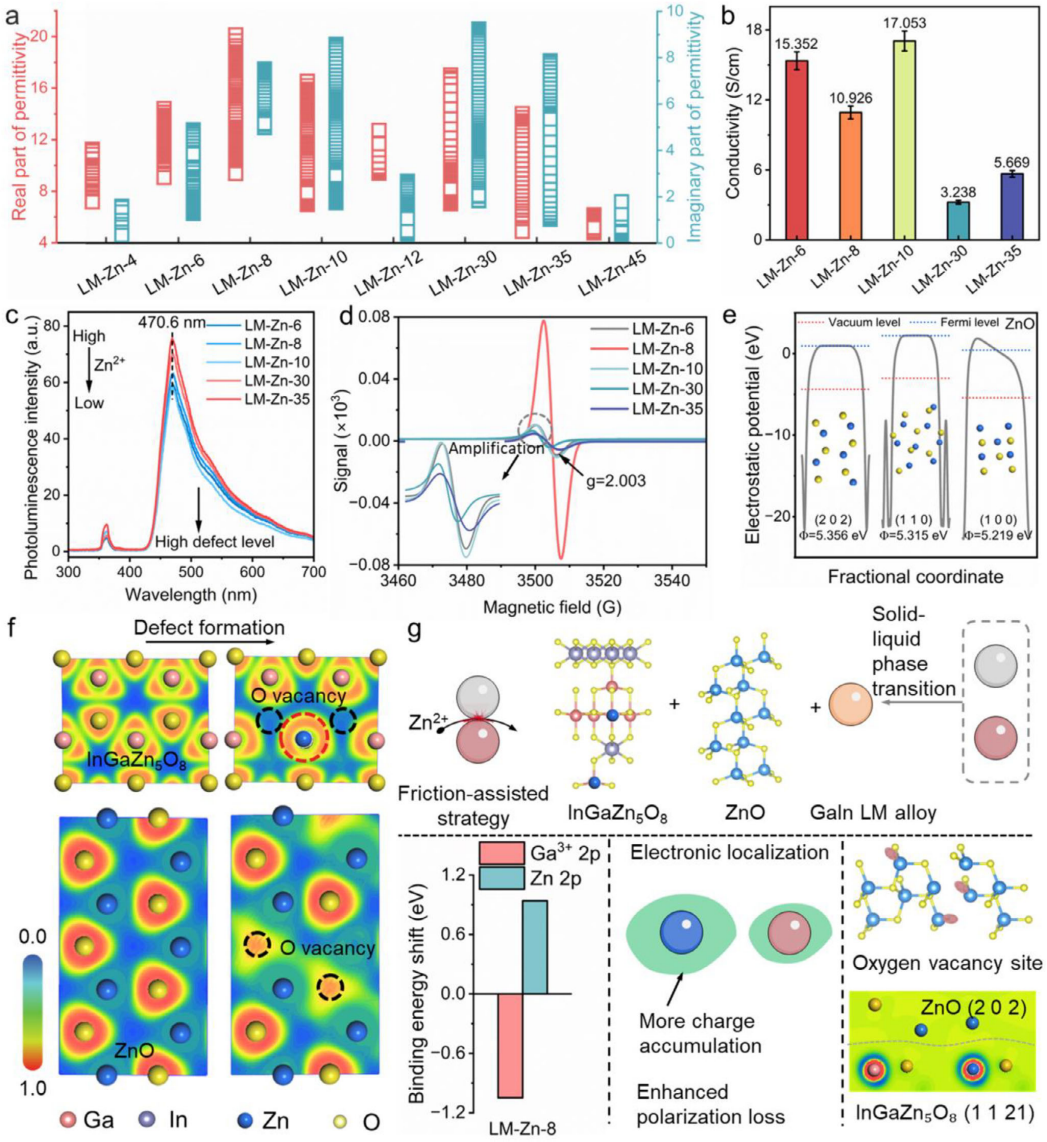
a) The complex permittivity of prepared samples with different Zn salt concentration; b) The intrinsic conductivity of LM‐Zn‐x (where x = 6, 8, 10, 30 and 35); c) Photoluminescence spectrum with an excitation wavelength of 375 nm; d) The EPR results of LM‐Zn system with different Zn salt concentration, showing that the LM‐Zn‐8 possesses the highest oxygen vacancy; e) The work functional of ZnO calculated via DFT; f) The electron localization function of InGaZn_5_O_8_ and ZnO with and without oxygen vacancy; g) Schematic illustration of EM loss mechanism in LM‐Zn.

Based on the aforementioned analyses, a systematic investigation was undertaken to evaluate the EM properties of LRP metal ions anchored via the friction‐assisted LM strategy, aiming to elucidate the underlying reduction and anchoring mechanisms for rational design of high‐performance EMW absorbers. Under a 70% filler loading ratio, the evolution of EM parameters with varying Zn salt concentrations reveals four distinct dielectric response modules (Figure S24, Supporting Information). The evolution of EM parameters with varying Zn salt concentrations reveals four distinct dielectric response modules (**Figure** **4**a, Figures S25 and S26, Supporting Information). Given the non‐magnetic nature (µ“≈1, µ“≈0) and the change in the loss tangent value of permeability is much smaller than that of permittivity, where magnetic loss contributions are negligible compared to dielectric dissipation (Figure S31, Supporting Information).^[^ ^48^, ^49^, ^50^ ^]^ This indicates that magnetic loss plays a secondary role in the overall EM loss mechanism, which is directly related to dielectric loss. The dielectric response exhibits four distinct concentration‐dependent regimes: i) In the low concentration range (LM‐Zn‐4 to LM‐Zn‐12), both the real and imaginary parts of permittivity initially increase, then decrease, forming relaxation peaks near 9 and 14 GHz. This behavior arises from Zn^2^⁺ substitution at Ga^3+^ sites, where the lower charge of Zn^2+^ weakens the Coulombic repulsion, promoting the electron cloud overlap and establishing localized dipole polarization centers around Zn/Ga sites. ii) With a further increase in Zn salt concentration, in the samples from LM‐Zn‐14 to LM‐Zn‐25, the EM parameters stabilize, with ε”≈10 and ε”≈0, accompanied by minor relaxation near 10 GHz. Here, the LM matrix percolation network is disrupted, suppressing charge transport and consequently diminishing conduction loss. iii) At 30 mmol Zn salt concentration, ε' rises from 10 (LM‐Zn‐25) to 17, accompanied by pronounced dielectric relaxation behavior as evidenced by *C*ole‐*C*ole semicircle analysis. Despite a threefold conductivity reduction (from 10.926 to 3.238 S cm^−1^) due to compromised percolative networks, ZnO/InGaZn_5_O_8_ heterointerfaces introduce WF mismatches, creating interfacial space charge regions that enhance polarization. iv) Excessive ZnO formation from Zn^2^⁺ and dissolved oxygen reactions leads to a sharp permittivity decline (ε′∼6, ε″≈1), attributed to the low dielectric response of ZnO aggregates. This concentration dependent dielectric regulation highlights the delicate balance between polarization enhancement and percolation conductive network integrity, providing a viewpoint for tailoring EM absorption.

To further explore the distinctive EM response of metal ions with LRP and quantify their contributions to conduction loss and polarization loss, we employed the four‐probe method to detect the intrinsic conductivity under different Zn salt concentrations, and quantitatively evaluated the respective contributions of conduction loss and polarization loss (Figure 4b and Figure S33, Supporting Information).^[^ ^51^ ^]^ Results showed that under lower Zn salt treatment, samples LM‐Zn‐6, LM‐Zn‐8, and LM‐Zn‐10 exhibited relatively high conductivities (15.352 S cm^−1^ for LM‐Zn‐6) (Figure S33, Supporting Information). Subsequently, the disruption of the LM percolation threshold conductive network significantly reduced the intrinsic conductivity to 3.238 S cm^−1^. Notably, polarization loss dominated the dissipation process, particularly in LM‐Zn‐8, where the maximum contribution of conduction loss was 0.98, while that of polarization loss reached 7.28, approximately seven times higher, indicating that polarization loss dominated in the LM‐Zn‐8 sample. Photoluminescence (PL, with an excitation wavelength of 375 nm) reveals a Zn salt concentration dependent defect density (Figures 4c and S34, Supporting Information), establishing a direct correlation between compositional variation and structural defects.^[^ ^52^ ^]^ Low concentrations induced substantial lattice distortion through Zn substitution/intercalation, enhancing polarization loss, while higher concentrations favored the formation of well‐ordered ZnO/InGaZn_5_O_8_ phases with minimal lattice strain/stress, as confirmed by GPA (Figure 4d,e). To further investigate the origin of defects, we used electron paramagnetic resonance (EPR) spectroscopy to detect the sensitivity of unpaired electron defects in the samples.^[^ ^53^ ^]^ The intensity of the uniaxial distortion signal at g = 2.003 was collected to evaluate the oxygen vacancy concentration, as shown in Figure 4d. The oxygen vacancy signal intensity in LM‐Zn‐8 was significantly higher than that in other samples, with an intensity eight‐fold greater than other samples. Abundant oxygen vacancies induce significant lattice strain/stress, thereby corroborating the GPA findings (Figure 2d,e). The accumulation of such vacancies disrupts charge homogeneity under alternating EM fields, directly triggering polarization behavior.^[^ ^54^, ^55^ ^]^ To further elucidate the EMW attenuation mechanism in friction‐assisted LM materials, we performed density functional theory (DFT) calculations on the system's electronic properties. InGaZn_5_O_8_ and ZnO exhibit distinct semiconductor characteristics with calculated band gaps of 1.606 and 0.704 eV, respectively (Figure S35, Supporting Information). This suitable electronic band structure effectively promoted charge migration under alternating electromagnetic fields. From HR‐TEM observations, we constructed crystallographic models of various facets (Figure 4e). The WF analysis reveals minimal variation across different crystal planes of homogeneous interface, suggesting the absence of significant interfacial charge accumulation at homojunction interfaces.^[^ ^27^ ^]^ Conversely, the heterostructure InGaZn_5_O_8_/ZnO exhibits a pronounced WF disparity, driving directional charge transfer from lower WF to higher one until Fermi level equilibration is achieved, indicating the presence of interface polarization in InGaZn_5_O_8_/ZnO heterogeneous interface. Based on HR‐TEM characterization, a heterogeneous InGaZn_5_O_8_ (1 1 21)/ZnO (2 0 2) interface was constructed. Differential charge density calculations reveal significant charge migration from ZnO to InGaZn_5_O₈ and accumulation on Zn/Ga sites.

When delving into the polarization loss mechanism, defects produced by the friction assisted strategy, as an important source of polarization, need to be thoroughly considered. The electron localization function (ELF) can reveal the localization phenomenon caused by defect formation in the lattice and vacancy, providing a theoretical basis for the breaking of charge symmetry. Given the high oxygen defect concentration level in EPR results, oxygen vacancies were introduced into the crystal structure to investigate localization charge distribution patterns (Figures 4f and S36, Supporting Information). In the InGaZn_5_O_8_ system, the introduction of oxygen vacancies did not significantly alter the local charge distribution characteristics, regardless of vacancy concentration. These defect sites fail to induce charge accumulation, whereas pronounced charge redistribution is observed specifically at Zn atomic sites. In contrast, Zn atoms in the ZnO system exhibited extremely low localization degrees, with electron cloud density primarily concentrated around O sites. When a high concentration of oxygen defects was introduced into this system, significant localized charge accumulation persisted in the oxygen defect regions. This differential localization effect reveals the asymmetric polarization mechanisms of Zn/O atom sites under alternating EM fields, providing a microstructural perspective for enhancing electromagnetic wave absorption performance.

Moreover, the EM attenuation mechanism of anchoring metal ions with LRP via the friction assisted strategy remains uninvestigated. The specific analysis of the response between the anchoring of LRP metals and EMW is as follows (Figure 4g). First, the friction‐assisted LM strategy overcomes persistent limitations in conventional MIRA—thermodynamic barriers, kinetic side‐reaction risks, and insufficient anchoring efficiency. Friction‐induced highly localized electric fields generate abundant free electrons, significantly lowering the reduction barrier for LRP ions. Concurrently, directional charge migration between Ga/In components enables specific capture by target LRP ions, suppressing parasitic pathways. Simultaneously, solid‐liquid phase transitions at the Ga/In interface create dynamically renewed active surfaces that maximize anchoring site utilization, thereby facilitating the formation of a distinct new phase. Second, the unique InGaZn_5_O_8_ crystal structure in LM‐Zn‐8 exhibits a characteristic positive Zn^2+^ 2p binding energy shift coupled with a negative Ga^3+^ 3d orbital shift. This heteroatomic substitution, enabled by the similar atomic radii of Zn and Ga, reduces local charge density, mitigating Coulombic repulsion while strengthening Zn‐O interactions (Figure 2g). The resultant asymmetry in charge distribution disrupts local charge symmetry, thereby enhancing interfacial polarization under alternating electric fields. Concurrently, EPR reveals high oxygen vacancy density, inducing lattice strain (validated by GPA, Figure 2d,e) that promotes electron scattering and dipolar polarization. Finally, DFT simulations confirm atomic‐scale charge localization: InGaZn_5_O_8_’s Zn ([Ar]3d^10^4s^2^) adopts a fully occupied electronic configuration, stabilizing localized charge accumulation near Zn sites with minimal delocalization, which synergistically intensifying polarization loss (Figure 4f). Concurrently, oxygen vacancies modulate charge aggregation within ZnO phases, creating abundant defect‐induced polarization sites. Both the crystal structures of InGaZn_5_O_8_ and ZnO exhibit distinct oxygen defect charge distributions, yet each demonstrates enhanced defect‐induced polarization. And mismatched WF, revealed by electrostatic potential calculations, drives space‐charge region formation at heterointerfaces, facilitating directional charge migration and interfacial charge redistribution (Figure 4e). Based on the mechanistic analysis above, concentration‐tuned engineering achieves a dual EAB band, establishing a universal paradigm for LRP‐based microwave absorbers.

## 3 Conclusion

In summary, we report a friction‐assisted liquid metal strategy enabling efficient MIRA for LRP ions. By harnessing dynamic interfaces at friction contacts, this approach generates high‐energy localized electric fields and directional charge transfer, overcoming persistent limitations in conventional MIRA, including thermodynamic barriers, parasitic kinetic pathways, and suboptimal anchoring efficiency. The dynamic LM interface facilitates effective Zn^2^⁺ anchoring, forming a distinct InGaZn_5_O_8_ phase. Experimental evidence (binding energy shifts, high oxygen vacancy concentration, lattice strain) establishes Zn/Ga substitution‐induced charge symmetry breaking and defect‐mediated polarization. Theoretical analyses confirm atomic‐scale charge localization at Zn sites and work function mismatch driven interfacial charge accumulation, synergistically enhancing polarization under alternating EM fields. The optimized LM‐Zn‐8 absorber achieves an exceptional 5.92 GHz EAB at 1.3 mm thickness (thickness‐normalized EAB: 4.55 GHz mm^−1^), outperforming established GaIn/GaInSn systems. The strategy demonstrates remarkable universality, extending to challenging LRP ions (Cr^3^⁺, Al^3^⁺) beyond conventional MIRA limits while exhibiting consistent concentration dependent dual EAB band behavior. This study establishes friction‐assisted MIRA as a universal platform and unveils the synthesis and absorption mechanisms for next‐generation microwave absorbers.

## Conflict of Interest

The authors declare no conflict of interest.

## Supporting information



Supporting Information

## Data Availability

The data that support the findings of this study are available from the corresponding author upon reasonable request.

## References

[1] Q. Zheng , J. Q. Wang , W. Q. Cao , H. Z. Zhai , M. S. Cao , Adv. Funct. Mater. 2025, 35, 2417972.

[2] K. Li , J. F. Wang , Q. Y. Shen , Y. Y. Song , R. Gao , Y. Wang , Adv. Energy Matter. 2024, 14, 2400956.

[3] Z. Q. Guo , D. Lan , C. H. Zhang , Z. G. Gao , M. Y. Han , X. T. Shi , M. K. He , H. Guo , Z. R. Jia , G. L. Wu , J. Mater. Sci. Technol. 2025, 220, 307.

[4] Y. H. Sun , J. X. Chen , X. M. Du , J. W. Cui , X. Chen , C. H. Wu , X. M. Yang , L. Q. Liu , J. H. Ye , Angew. Chem., Int. Ed. 2024, 63, 202410802. 10.1002/anie.202410802 38923695

[5] Q. X. Zhang , J. A. Wang , Q. H. Yu , Q. Z. Li , R. Z. Fan , C. Li , Y. Y. Fan , C. Zhao , W. H. Cheng , P. Y. Ji , J. Sheng , C. H. Zhang , S. H. Xie , G. Henkelman , H. Li , Nat. Synth. 2025, 4, 252.

[6] E. Satheeshkumar , T. Makaryan , A. Melikyan , H. Minassian , Y. Gogotsi , M. Yoshimura , Sci. Rep. 2016, 6, 32049. 27557838 10.1038/srep32049 PMC4997347

[7] Y. Liu , A. Xu , J. H. Wang , F. Y. Jiang , H. Peng , J. Yang , Y. L. Zhou , ACS Nano 2024, 18, 33197. 39568212 10.1021/acsnano.4c12188

[8] W. Q. Song , C. X. Xiao , J. Ding , Z. C. Huang , X. Y. Yang , T. Zhang , D. Mitlin , W. B. Hu , Adv. Mater. 2024, 36, 2301477. 10.1002/adma.202301477 37078970

[9] C. C. Cai , X. Y. Li , P. Hu , T. Zhu , J. T. Li , H. Fan , R. H. Yu , T. Y. Zhang , S. Lee , L. Zhou , L. Q. Mai , Adv. Funct. Mater. 2023, 33, 2215155.

[10] S. G. Ji , M. M. Kim , M. H. Han , J. Cho , Y. Son , Y. Y. Kim , J. Jeong , Z. H. Kim , K. H. Chae , H. S. Oh , H. Kim , C. H. Choi , Nat. Catal. 2024, 7, 1330.

[11] X. F. Zhou , Y. Liu , Z. J. Gao , P. Min , J. Liu , Z. Z. Yu , V. Nicolosi , H. B. Zhang , Adv. Mater. 2024, 36, 2310849. 10.1002/adma.202310849 38185468

[12] J. J. Zheng , S. D. Mao , S. J. Zhang , J. Liu , Y. Y. Song , S. Y. Zhang , Y. P. Jiao , S. H. Zhang , X. Q. Huang , D. Lan , G. L. Wu , J. Alloy. Compd. 2025, 1022, 179884.

[13] J. M. Tang , J. B. Tang , M. Mayyas , M. B. Ghasemian , J. Sun , M. A. Rahim , J. Yang , J. L. Han , D. J. Lawes , R. Jalili , T. Daeneke , M. G. Saborio , Z. B. Cao , C. A. Echeverria , F. M. Allioux , A. Zavabeti , J. Hamilton , V. Mitchell , A. P. O'Mullane , R. B. Kaner , D. Esrafilzadeh , M. D. Dickey , K. K. Zadeh , Adv. Mater. 2022, 34, 2105789. 10.1002/adma.202105789 34613649

[14] H. H. Luo , L. Y. Zhang , H. Q. Yang , W. Yang , Q. J. Liu , W. H. Mu , K. Boudmyxay , J. Liu , P. Z. Yang , L. F. Duan , Adv. Funct. Mater. 2024, 35, 2413156.

[15] W. Q. Peng , Y. Zhang , Z. J. Zhang , H. Zhao , H. H. Huang , J. M. Zhao , B. X. Cheng , J. X. He , B. Xu , B. J. Shang , S. X. Nie , S. F. Wang , Q. S. Duan , Nano Lett. 2025, 25, 6622. 40208821 10.1021/acs.nanolett.5c00665

[16] L. Ren , N. Y. Cheng , X. K. Man , D. C. Qi , Y. D. Liu , G. B. Xu , D. D. Cui , N. N. Liu , J. X. Zhong , G. Peleckis , X. Xu , S. X. Dou , Y. Du , Adv. Mater. 2021, 33, 2008024. 10.1002/adma.202008024 33522010

[17] B. Zhao , Y. Q. Du , H. L. Lv , Z. K. Yan , H. Jian , G. Y. Chen , Y. Y. Wu , B. B. Fan , J. C. Zhang , L. M. Wu , D. W. Zhang , R. C. Che , Adv. Funct. Mater. 2023, 33, 2302172.

[18] H. J. Hwang , H. Hong , B. G. Cho , H. K. Lee , J. S. Kim , U. J. Lee , W. Kim , H. Kim , K. B. Chung , D. Choi , Nano Energy 2021, 90, 106647.

[19] Q. H. Tang , S. H. Han , M. J. Yao , D. J. Singh , J. Y. Xi , H. J. Liu , J. Yang , Energy Environ. Sci. 2024, 17, 611.

[20] S. M. Liu , Y. Liu , S. H. Wang , L. Lin , Y. S. Zhou , Y. F. Hu , Z. L. Wang , Adv. Mater. 2013, 25, 6184. 24038597 10.1002/adma.201302808

[21] X. Chen , X. Peng , C. H. Wei , Z. X. Wang , J. He , H. R. Sheng , T. Jiang , K. Dong , Adv. Funct. Mater. 2025, 35, 2415421.

[22] H. Hiramatsu , K. Ueda , H. Ohta , T. Kamiya , M. Hirano , H. Hosono , Appl. Phys. Lett. 2005, 87, 211107.

[23] L. X. Fang , B. L. Zhang , W. Li , X. J. Li , T. J. Xin , Q. Y. Zhang , RCS Adv. 2014, 4, 7167.

[24] G. Chen , T. Zhang , L. M. Zhang , K. Tao , Q. Chen , H. J. Wu , Mater. Horiz. 2025, 12, 1629. 39660567 10.1039/d4mh01564a

[25] Y. J. Luan , X. L. Yan , C. W. Ji , C. P. Lv , D. Lan , S. Y. Zhang , J. Sun , D. H. Li , G. L. Wu , J. Mater. Sci. Technol. 2025, 228, 279.

[26] M. Na , V. Singh , R. H. Choi , B. G. Kim , H. R. Byon , Energy Storage Mater. 2023, 57, 195.

[27] S. C. Hui , Q. Chen , K. Tao , L. M. Zhang , X. M. Fan , R. C. Che , H. J. Wu , Adv. Mater. 2025, 37, 2415844. 10.1002/adma.202415844 39593259

[28] R. Z. Xing , G. X. Xu , N. Qu , R. Zhou , J. Y. Yang , J. Kong , Adv. Funct. Mater. 2024, 34, 2307499.

[29] F. Zhang , X. C. Sun , M. Du , X. F. Zhang , W. T. Dong , Y. H. Sang , J. J. Wang , Y. L. Li , H. Liu , S. H. Wang , Energy Environ. Mater. 2021, 4, 620.

[30] W. Q. Cao , M. Zhang , M. S. Cao , Adv. Funct. Mater. 2024, 34, 2410928.

[31] D. Wu , D. Lan , S. J. Zhang , Q. C. He , X. P. Zhou , Y. Q. Wang , Mater. Today Nano 2024, 28, 100520.

[32] K. Xie , Q. Zhang , F. Chen , Q. Fu , J. Mater. Chem. A 2025, 13, 1887.

[33] X. F. Zhang , L. L. Xu , J. T. Zhou , W. J. Zheng , H. Jiang , K. Zuraiqi , G. K. Li , J. Liu , A. Zavabeti , ACS Appl. Nano Mater. 2021, 4, 9200.

[34] X. F. Zhang , H. Jiang , L. L. Xu , K. Zuraiqi , T. Daeneke , J. T. Zhou , G. K. Li , A. Zavabeti , Ceram. Int. 2022, 48, 10066.

[35] L. C. Wang , L. Huang , Y. B. Li , Y. Yuan , J. Appl. Phys. 2022, 132, 194101.

[36] Y. Wang , Y. N. Gao , T. N. Yue , X. D. Chen , R. C. Che , M. Wang , J. Colloid Interf. Sci. 2022, 607, 210. 10.1016/j.jcis.2021.08.206 34500420

[37] X. F. Zhang , Z. Y. Wang , L. L. Xu , K. Zuraiqi , T. Daeneke , Z. J. Yao , D. C. Qi , A. Zavabeti , J. Colloid Interf. Sci. 2022, 606, 1852. 10.1016/j.jcis.2021.08.143 34507176

[38] K. Xie , Q. Zhang , F. Chen , Q. Fu , J. Mater. Chem. C 2025, 13, 7167.

[39] K. Y. Zhao , C. L. Luo , C. Sun , M. L. Huang , M. Wang , Compos. Part A‐Appl. S. 2023, 173, 107640.

[40] Y. L. Li , J. C. Liu , D. S. Li , M. M. Fu , A. M. Xie , W. J. Li , H. Liang , Y. G. Li , X. Chen , C. Yu , Rare Met. 2025, 44, 3299.

[41] B. Zhao , Z. K. Yan , L. L. Liu , Y. Y. Zhang , L. Guan , X. Q. Guo , R. S. Li , R. C. Che , R. Zhang , Adv. Funct. Mater. 2024, 34, 2314008.

[42] Z. Z. Wang , Q. Zheng , M. J. Yu , M. S. Cao , J. Mater. Sci. Technol. 2025, 228, 1.

[43] X. T. Sun , Z. Wu , X. L. Tan , Y. Q. Xing , P. Huang , B. J. Li , L. Liu , Carbon 2025, 233, 119909.

[44] J. Y. Zong , H. Z. Zhai , H. Z. Guan , Z. Z. Wang , M. S. Cao , W. Q. Cao , Adv. Funct. Mater. 2025, 10.1002/adfm.202507277.

[45] X. J. Liu , Q. Wang , J. Cui , Y. H. Yan , Carbon 2025, 233, 119860.

[46] X. Y. Sun , X. C. Li , P. A. Chen , Y. L. Zhu , Carbon 2025, 234, 119963.

[47] S. Xu , Q. H. Liu , Z. L. Yu , X. D. Dai , S. Y. Yu , C. J. Li , Carbon 2025, 234, 119859.

[48] J. Y. Zong , M. S. Cao , Mater. Today Phys. 2024, 43, 101400.

[49] L. Y. Li , M. Zhang , M. Jiang , L. H. Gao , Z. Ma , M. S. Cao , Adv. Funct. Mater. 2025, 35, 2416673.

[50] A. L. Feng , L. Y. Yu , D. Lan , C. P. Lv , S. Y. Zhang , Z. G. Gao , Z. Q. Guo , G. L. Wu , J. Mater. Sci. Technol. 2025, 228, 225.

[51] J. M. Wen , Y. L. Liu , S. C. Hui , L. C. Deng , L. M. Zhang , X. M. Fan , Q. Chen , X. M. Liu , X. C. Li , N. Yan , H. J. Wu , Matter 2025, 8, 102151.

[52] K, Y. , F. Pan , H. S. Liang , X. Zhang , L. X. Li , L. X. Song , Y. Yang , B. Yuan , W. Lu , Adv. Funct. Mater. 2025, 35, 2413639.

[53] R. W. Feng , D. Lan , Y. Q. Li , Y. C. He , Q. C. He , Y. Q. Wang , Ceram. Int. 2024, 50, 55461.

[54] M. Y. Cui , Y. Wei , S. C. Hui , T. Zhang , G. Chen , Y. Zhang , S. Y. Zhang , Z. G. Gao , J. Q. Zhang , H. J. Wu , Adv. Funct. Mater. 2025, 10.1002/adfm.202508939.

[55] S. C. Hui , X. Zhou , L. M. Zhang , H. J. Wu , Adv. Sci. 2024, 11, 2307649. 10.1002/advs.202307649 PMC10853738 38044282

