# Supplementary material for: Friction‐Assisted Liquid Metal‐Driven Anchoring of Low Redox Potential Metal Ions for Enhanced Electromagnetic Wave Absorption
Source: Adv Sci (Weinh). 2025 Aug 11;12(41):e11810. doi: 10.1002/advs.202511810 (PMC12591199; doi:10.1002/advs.202511810)
Supplement: Supplementary file 1 — Supporting Information [file ADVS-12-e11810-s001.docx]

Supporting Information for

**Friction-Assisted Liquid Metal-Driven Anchoring of Low Redox Potential Metal Ions for Enhanced Electromagnetic Wave Absorption**

Tao Zhang^1, 2^, Geng Chen^2^, Lechun Deng^3^, Limin Zhang^2, *^, Hao Shen^4, *^, Qiang Chen^3^, Hu Liu^1, *^, Hongjing Wu^1, 2, *^

^1^ Key Laboratory of Green and High-end Utilization of Salt Lake Resources, Qinghai Engineering and Technology Research Center of Comprehensive Utilization of Salt Lake Resources, Qinghai Institute of Salt Lakes, Chinese Academy of Sciences, Xining 810008, China;

^2^ MOE Key Laboratory of Material Physics and Chemistry under Extraordinary Conditions, School of Physical Science and Technology, Northwestern Polytechnical University, Xi’an 710072, China;

^3^ State Key Laboratory of Solidification Processing, School of Materials Science and Engineering, Northwestern Polytechnical University, Xi’an 710072, China;

^4^ Department of Applied Physics, School of Science, Chang’an University, Xi’an 710064, China;

^*^ Corresponding authors: [liminzhang@nwpu.edu.cn](mailto:liminzhang@nwpu.edu.cn) (L. Zhang); [shenhao@chd.edu.cn](mailto:shenhao@chd.edu.cn) (H. Shen); [liuhu@isl.ac.cn](mailto:liuhu@isl.ac.cn) (H. Liu); wuhongjing@mail.nwpu.edu.cn (H. Wu)

**This file includes:**

Supplementary Text

Figure S1 to S33

Table S1 to S2

References

**Supplementary Text**

**Experimental section**

**Raw Materials**

Zinc nitrate hexahydrate (Zn(NO_3_)_2_⋅6H_2_O), Chromium nitrate nonahydrate (Cr(NO_3_)_3_⋅9H_2_O), Aluminum nitrate nonahydrate (Al(NO_3_)_3_⋅9H_2_O), solid gallium particles, solid indium particles, and deionized water were purchased from Aladdin Industrial Corporation (Shanghai, China) and used as received without further purification.

**Synthesis of LM-Zn with different Zn^2+^ concentration**

Gallium and indium particles with a mass ratio of 3:1 were weighed and thoroughly mixed, then placed in a mixed solution of deionized water and ethanol at a volume ratio of 2:1. Subsequently, zinc nitrate with different molar ratios was added to the above solution. The entire solution was transferred into an ultrasonic processor, ensuring the ultrasonic probe was completely submerged. Ultrasonication was performed at 80% power with a pulsed mode (10 s on, 3 s off) for 1 h. Finally, the resulting mixture was centrifuged, washed, and dried to obtain the samples, which were named LM-Zn-x according to the concentration of Zn^2+^, where x denotes the molar amount.

**Synthesis of LM-Cr with different Cr^3+^ concentration**

The synthesis process of LM-Cr is similar to that of LM-Zn, with only the type of metal salt being changed.

**Synthesis of LM-Al with different Al^3+^ concentration**

The synthesis process of LM-Al is similar to that of LM-Zn, with only the type of metal salt being changed.

**Characterization methods**

**Materials Characterization**

The phase compositions of all samples were characterized by X-ray diffraction (XRD, Cu Kα radiation) with a scanning range of 20°-90°. Microscopic surface morphologies and crystal angles were observed via scanning electron microscopy (SEM, ZEISS Sigma 300, Germany). Transmission electron microscopy (TEM, FEI Talos F200x, USA) was used to analyze the lattice structures, with samples first dispersed in ethanol via sonication and then deposited onto copper meshes. Chemical state characterizations were performed by X-ray photoelectron spectroscopy (XPS, Kratos Axis Ultra DLD, UK). Photoluminescence (PL) spectroscopy quantified defect concentrations, while intrinsic conductivity of powder samples was measured using a four-probe resistivity meter (ST2742 B) under 30 MPa pressure.

**Electromagnetic Measurement**

Electromagnetic parameters (permittivity ε', ε'' and permeability μ', μ'') of all samples were measured using a network vector analyzer (Anritsu MS46322B, Japan) across 2–18 GHz. Samples were mixed with paraffin at 70% mass ratios for coaxial testing (outer diameter 7.00 mm, inner diameter 3.04 mm). Reflection loss (RL) was used to evaluate electromagnetic wave (EMW) absorption performance, with RL < -10 dB indicating 90% energy absorption. RL was calculated using transmission line theory [S1]:

$RL=20\log\left| \frac{Z_{in}-Z_{0}}{Z_{in}+Z_{0}} \right|$ (1)

$Z_{in}=Z_{0}\sqrt{\frac{\mu_{r}}{\varepsilon_{r}}} tanh\left( j\frac{2\pi fd}{c}\sqrt{\mu_{r}\varepsilon_{r}} \right)$ (2)

Among them, Z_in_, Z_0_, c and d refer to the impedance of the absorber, impedance of free space, velocity of light and the thickness of absorber, respectively.

Among them, Z_0_, Z_in_, d and c refer to the impedance of free space, impedance of the absorber, the thickness of absorber, and velocity of light, respectively.

**Radar cross section (RCS) simulation**

RCS simulations were conducted using CST software based on far-field response. The model comprised an upper wave-absorbing layer and a lower perfect electrical conductor (PEC) layer (1 mm thickness), placed in the XOY plane with plane waves incident along the negative Z-axis and electric polarization along the Z-axis. Open boundary conditions were applied, and the field was monitored at 10 GHz. RCS performance was evaluated using the equation [S2]:

$\mathrm{RCS}\left( \mathrm{dB}m^{2} \right)=10log\left( \frac{4S}{{}^{2}}\left| \frac{E_{S}}{E_{i}} \right|^{2} \right)$ (3)

Where *S* is the simulated plate area (100 mm × 100 mm), *λ* is the EM wave wavelength, and *E_S_*​ and *E_i_*​denote scattered and incident electric field intensities, respectively.

**Conduction loss (ε_c_″) and polarization loss (ε_p_″) calculation**

The 𝜀_c_″ and 𝜀_p_″ can be calculated into the following equations [S3]:

$={}_{c}+{}_{p}=\frac{{}_{s}-{}}{1+{}^{2}{}^{2}}+\frac{}{{}_{0}}$ (4)

${}_{c}=\frac{}{{}_{0}}$ (5)

**Density functional theory (DFT) calculation**

Differential charge density calculations were performed based on density functional theory (DFT). Work function calculations, along with DFT band structure and density of states analyses, were conducted using the Cambridge Serial Total Energy Package (CASTEP) module in Materials Studio. The Perdew-Burke-Ernzerhof (PBE) functional within the generalized gradient approximation (GGA) framework was used for exchange-correlation effects, with Monkhorst-Pack K-point sampling of 3×2×1 applied throughout. Structures were geometrically optimized using the conjugate gradient method until energy convergence below 10^-5^ eV was achieved. For work function calculations, periodic boundary conditions were applied in all directions, with a 15 Å vacuum layer along the z-axis to separate periodic slabs.

**Supplementary Figures**

**
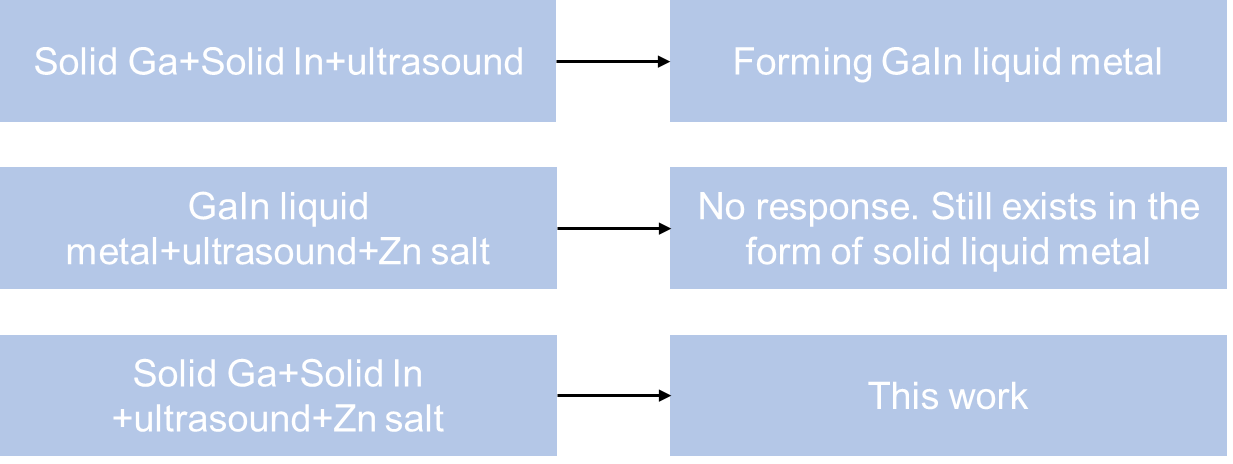
**

**Figure S1.** A series of contrast experiments.


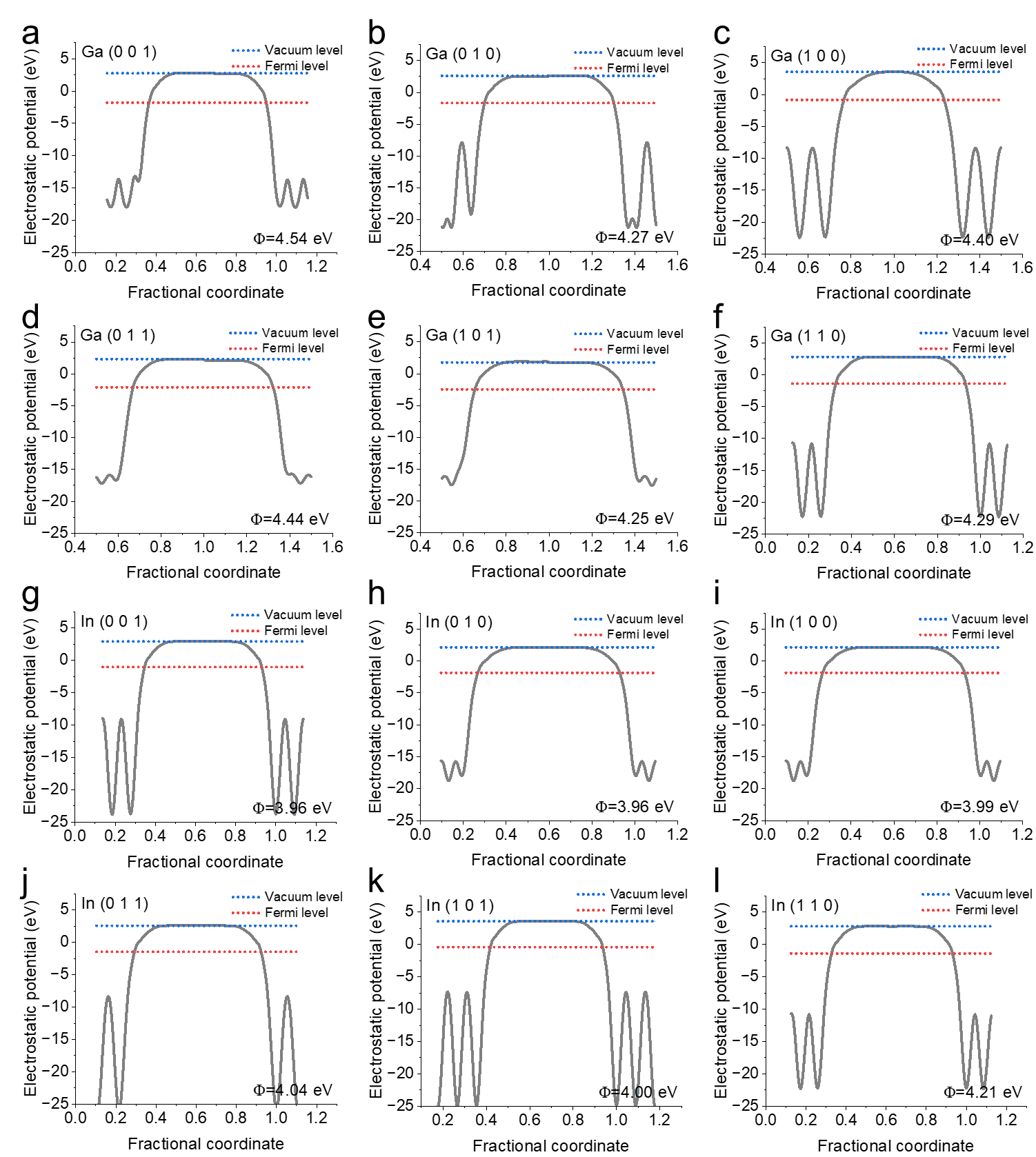


**Figure S2** Calculated values of the work function for different crystal planes of Ga and In using the first-principle.


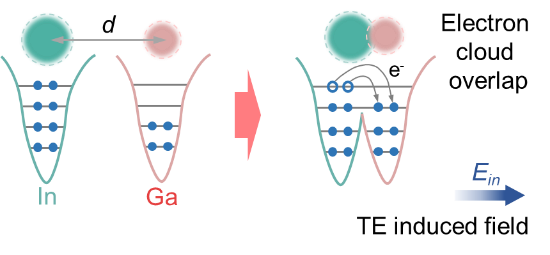


**Figure S3.** Constructed electron-cloud potential-well model explains the contact initiation mechanism between Ga and In.


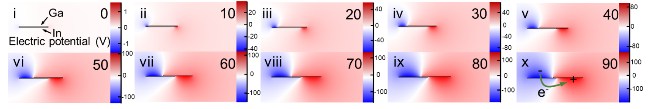


**Figure S4.** Simulation of surface potential distribution from friction on Ga/In surfaces using COMSOL Multiphysics software.


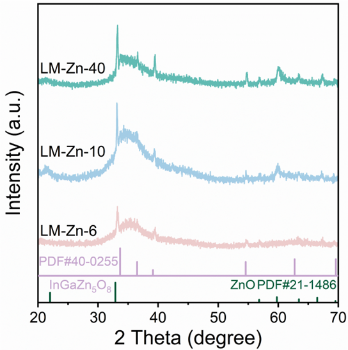


**Figure S5.** The XRD patterns of LM-Zn-6, LM-Zn-10 and LM-Zn-40.


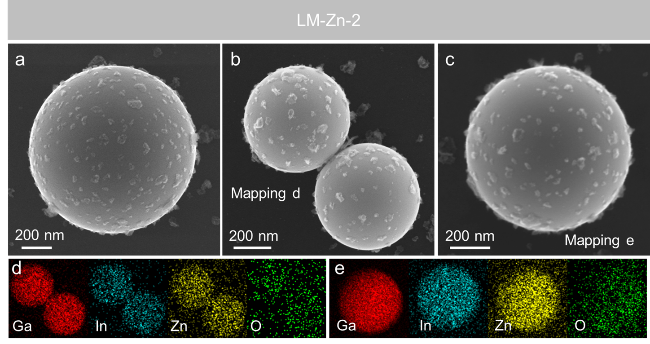


**Figure S6.** The SEM image of LM-Zn-2.


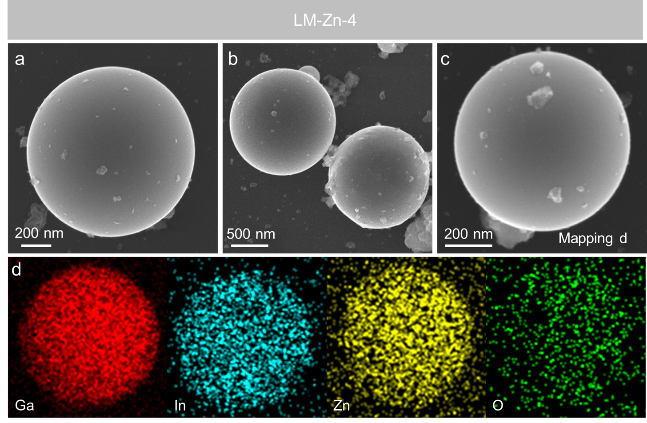


**Figure S7.** The SEM image of LM-Zn-4.


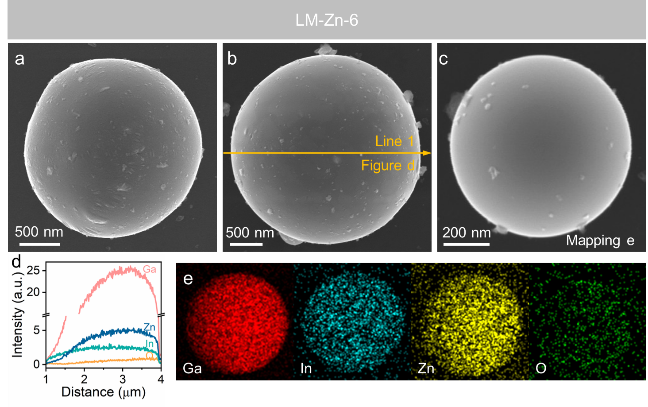


**Figure S8.** The SEM image of LM-Zn-6.


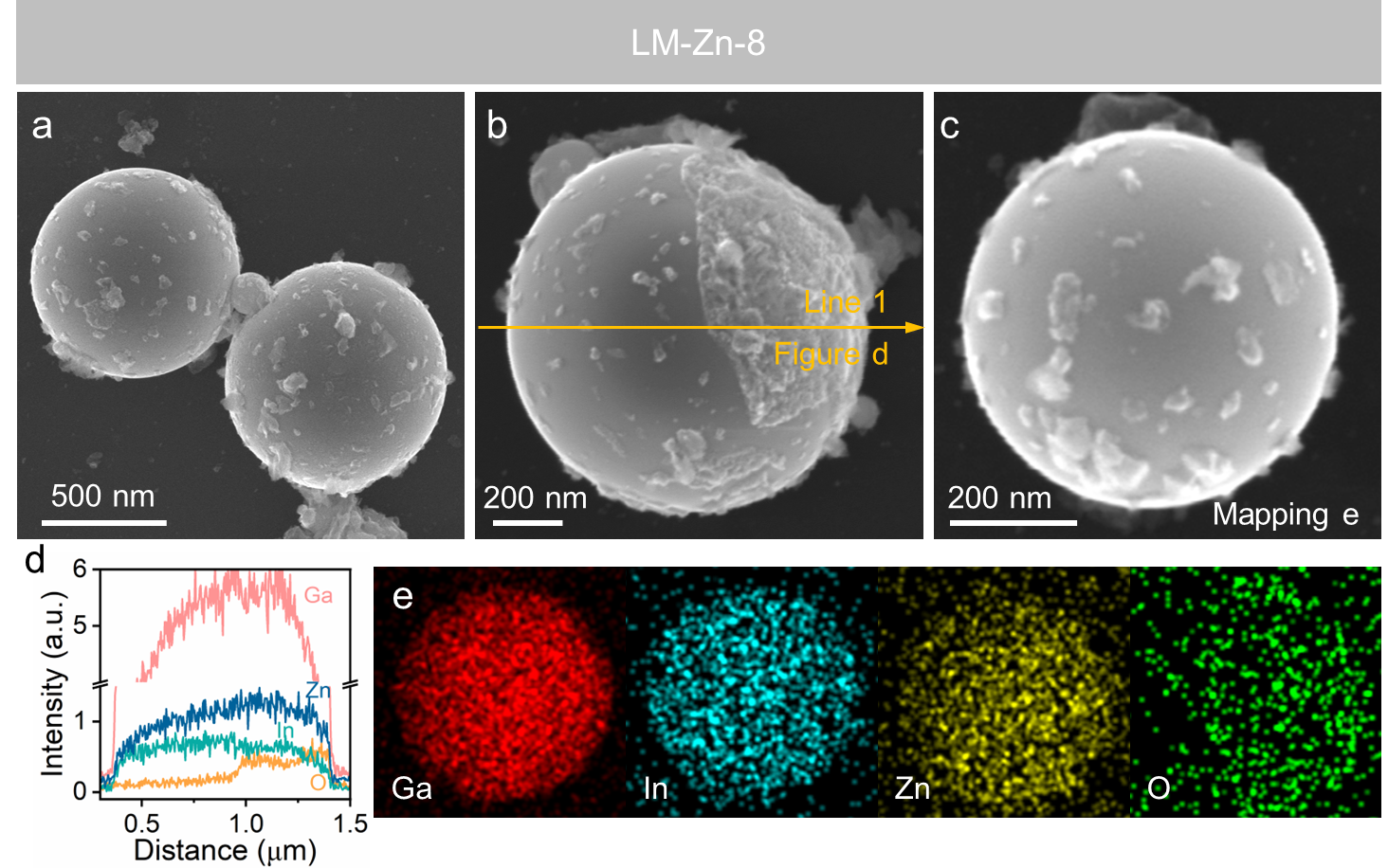


**Figure S9.** The SEM image of LM-Zn-8.


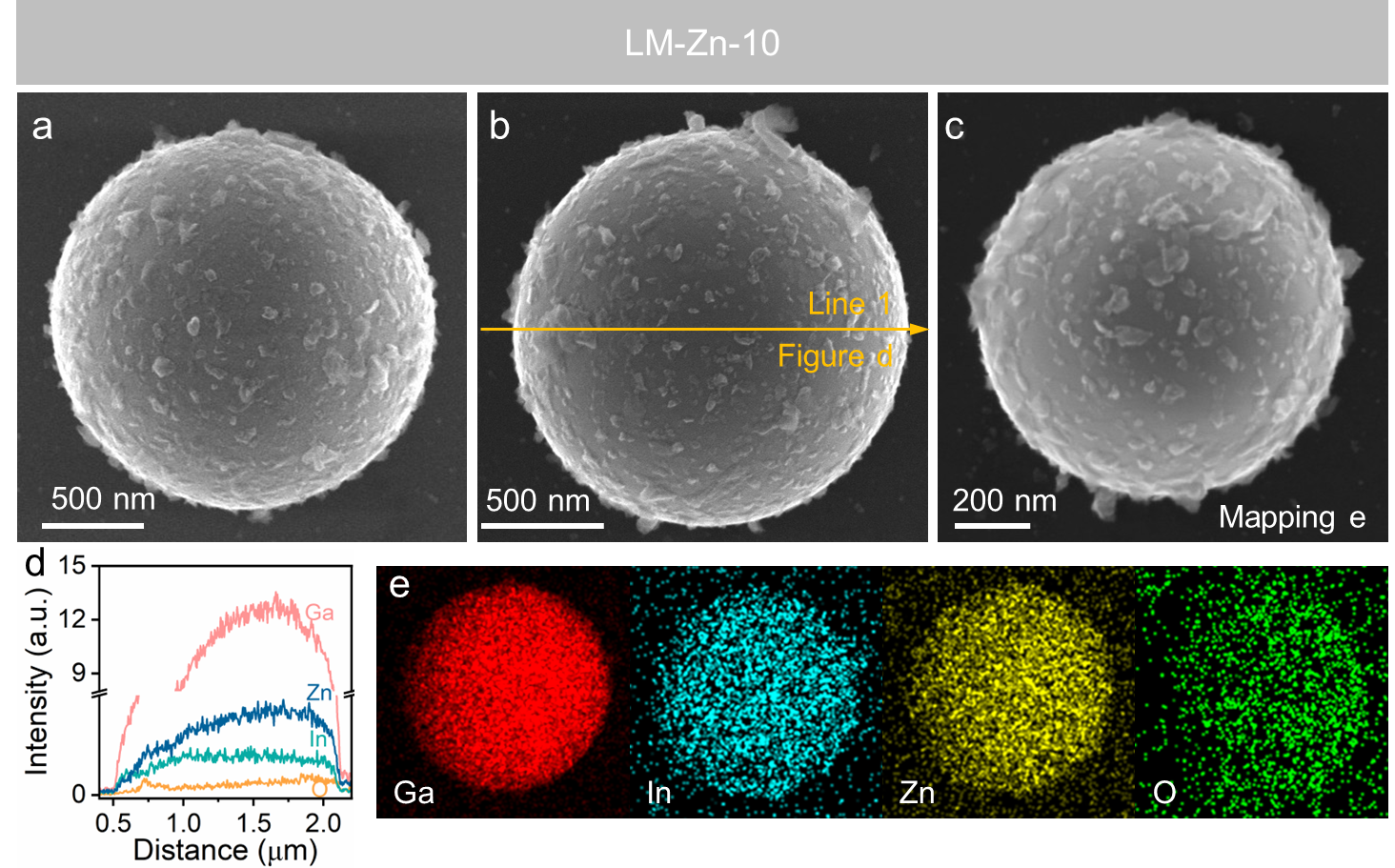


**Figure S10.** The SEM image of LM-Zn-10.


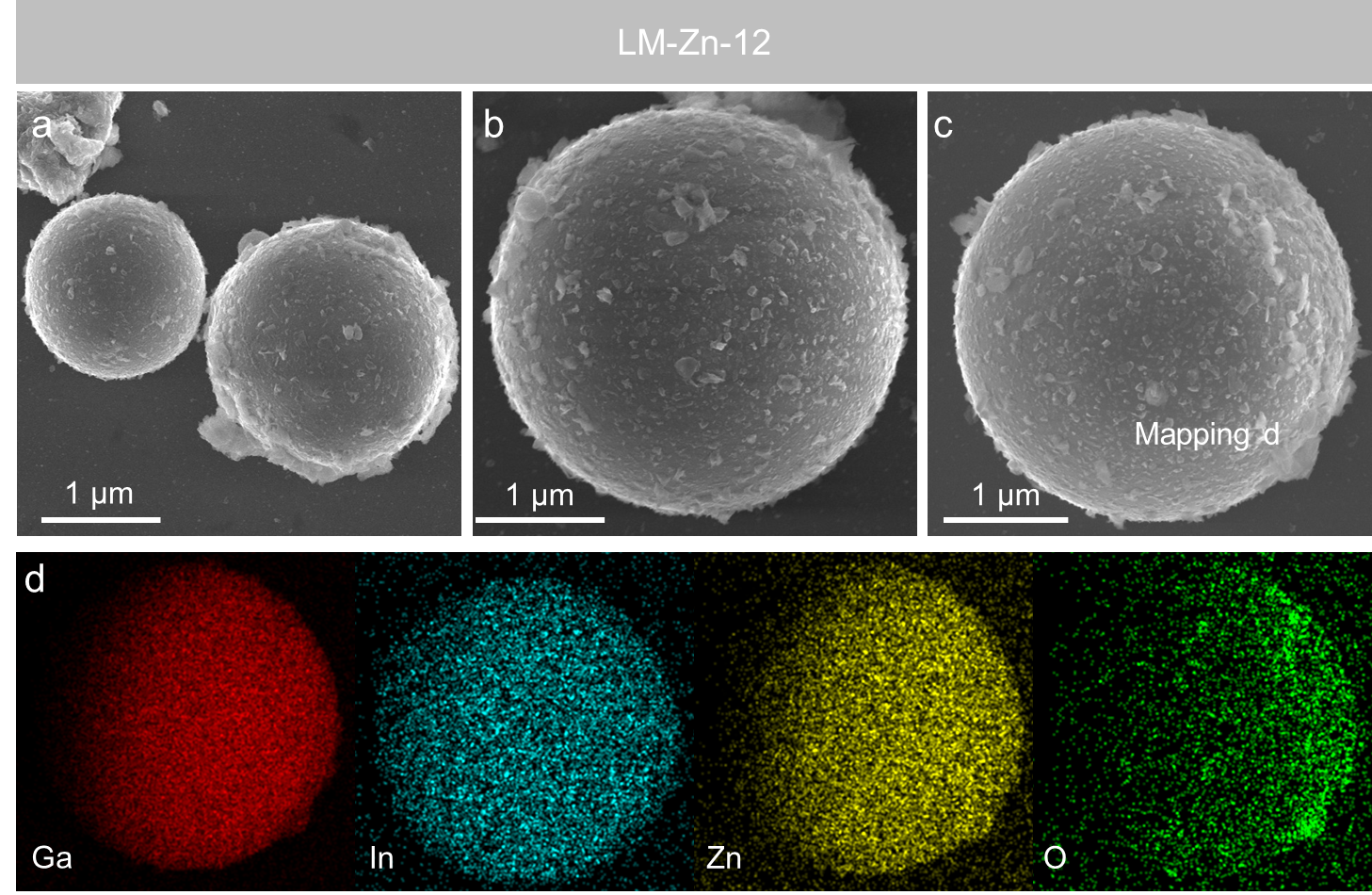


**Figure S11.** The SEM image of LM-Zn-12.


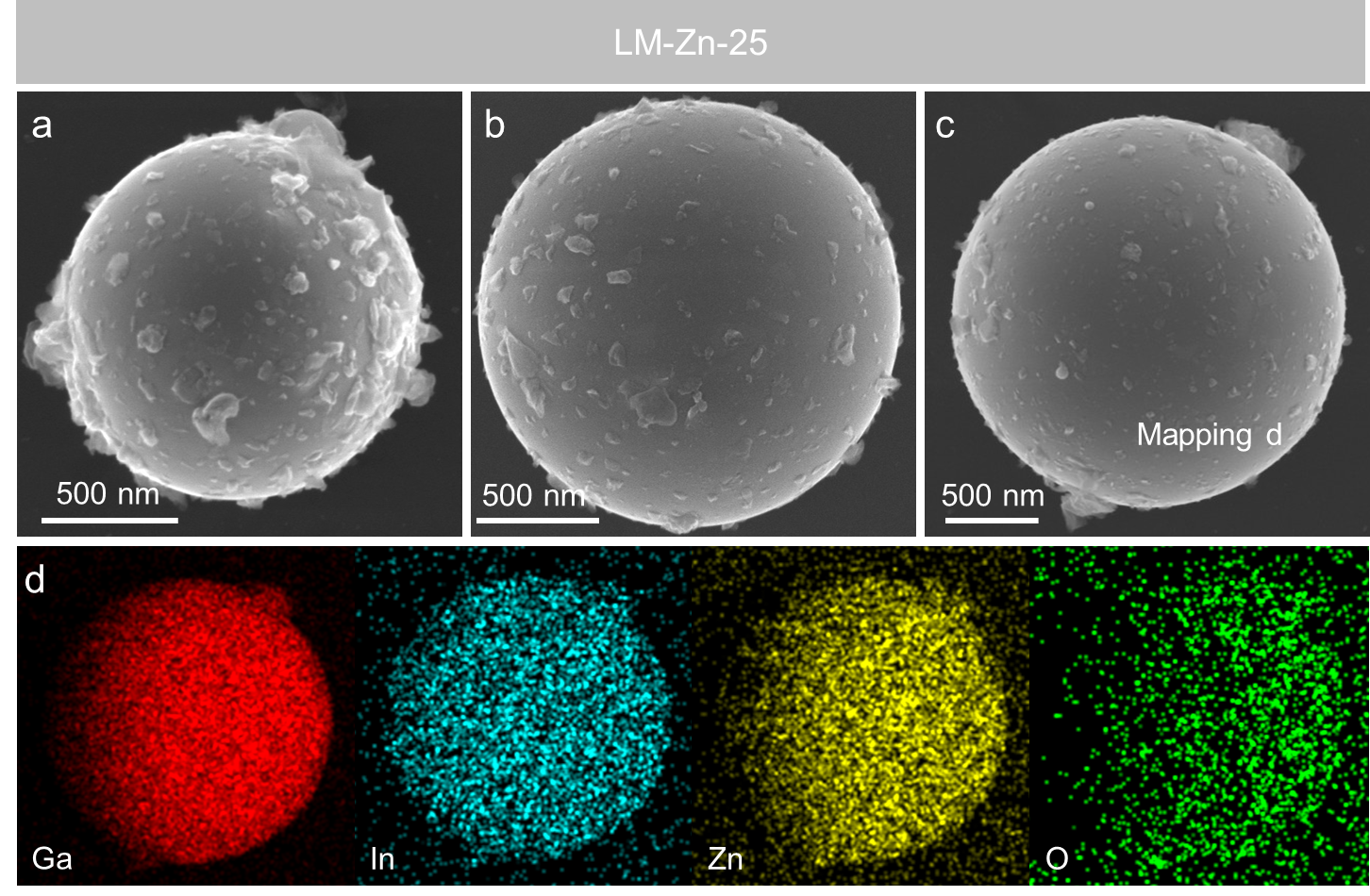


**Figure S12.** The SEM image of LM-Zn-25.


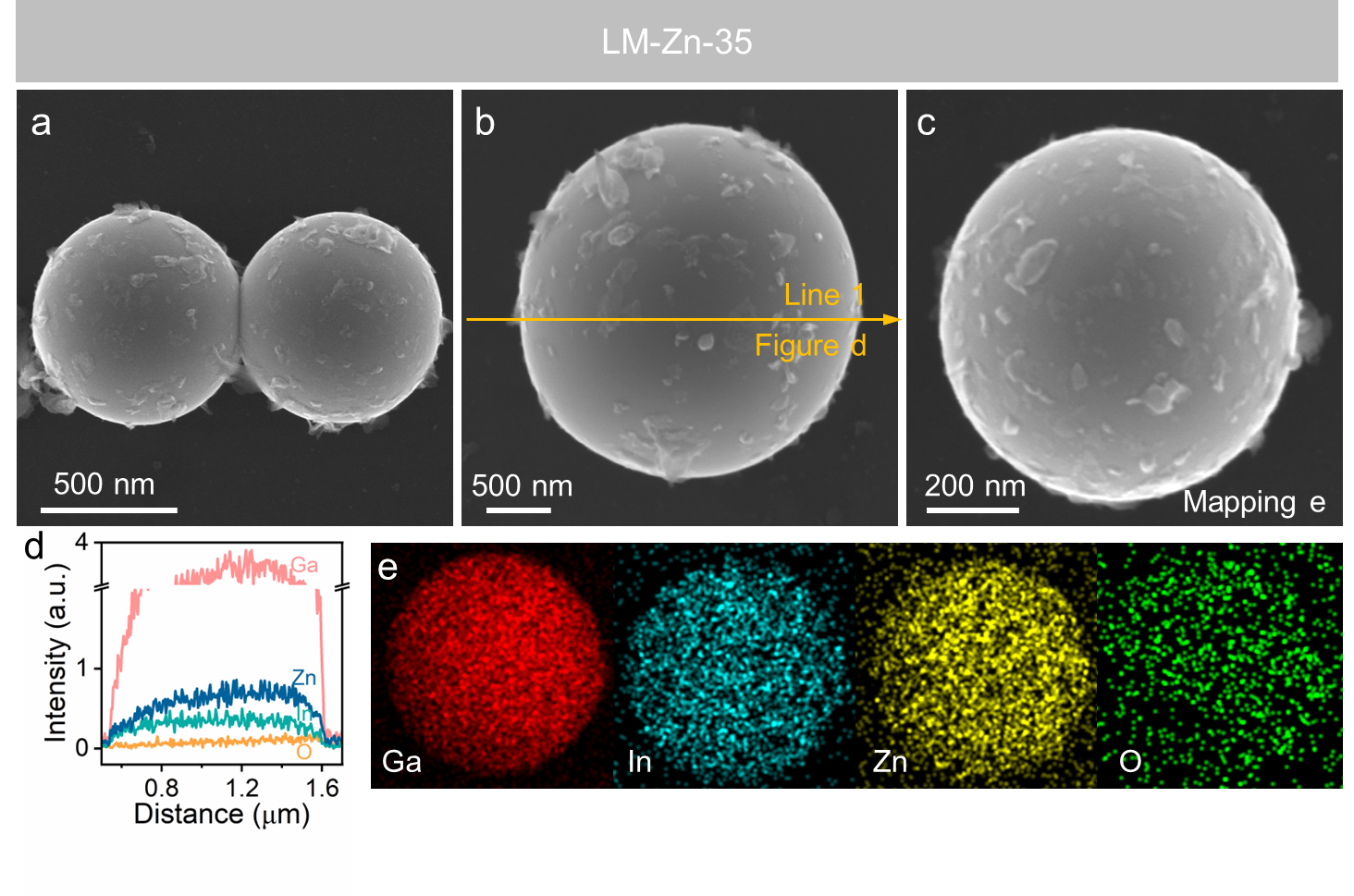


**Figure S13.** The SEM image of LM-Zn-35.


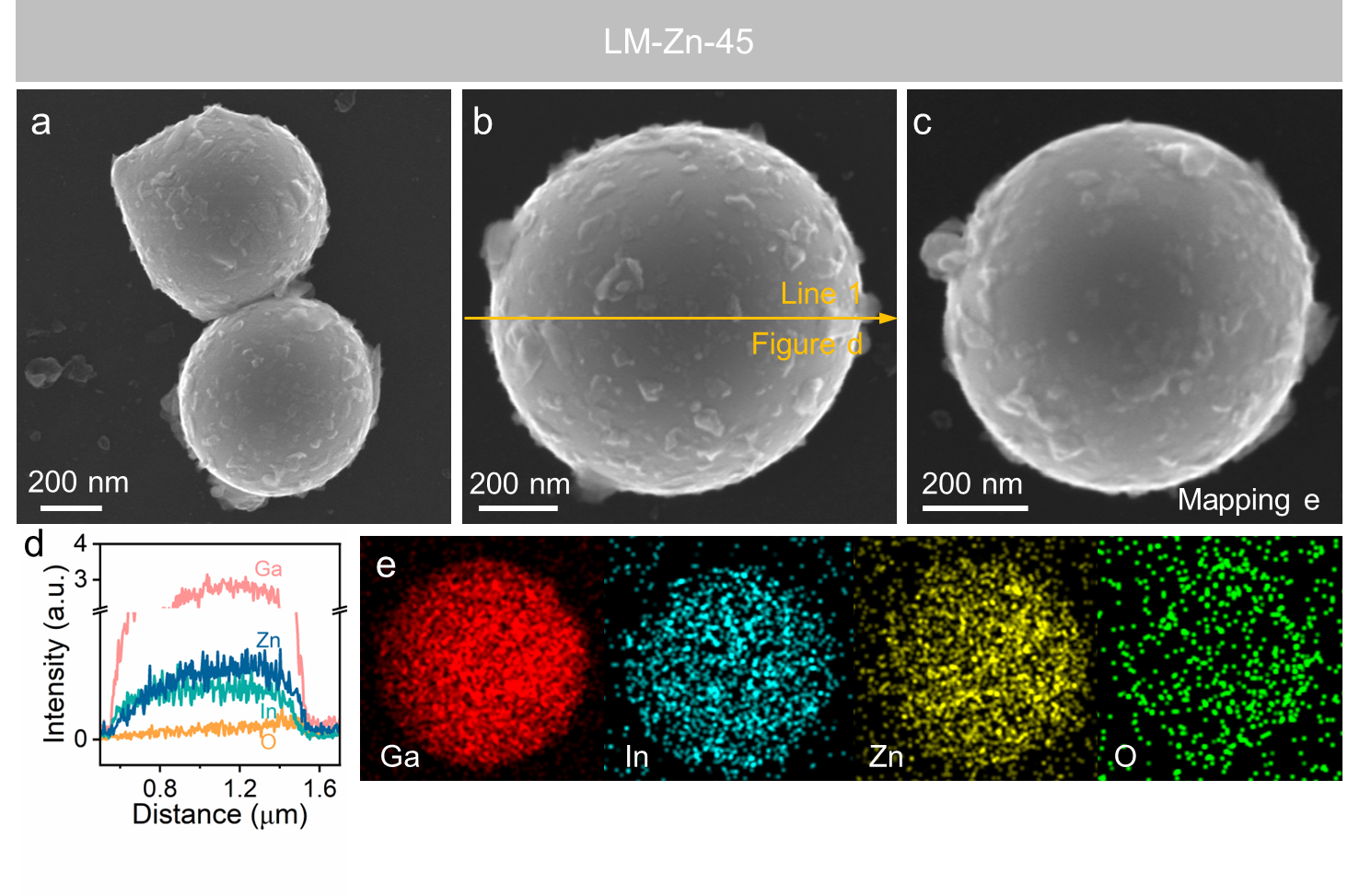


**Figure S14.** The SEM image of LM-Zn-45.


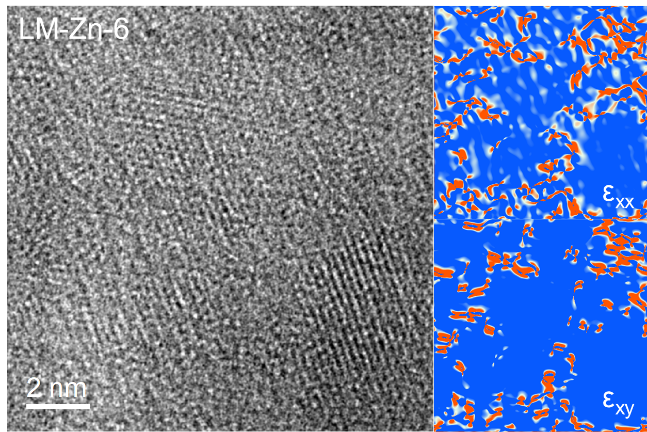


**Figure S15.** The TEM image of LM-Zn-6 and corresponding GPA images.


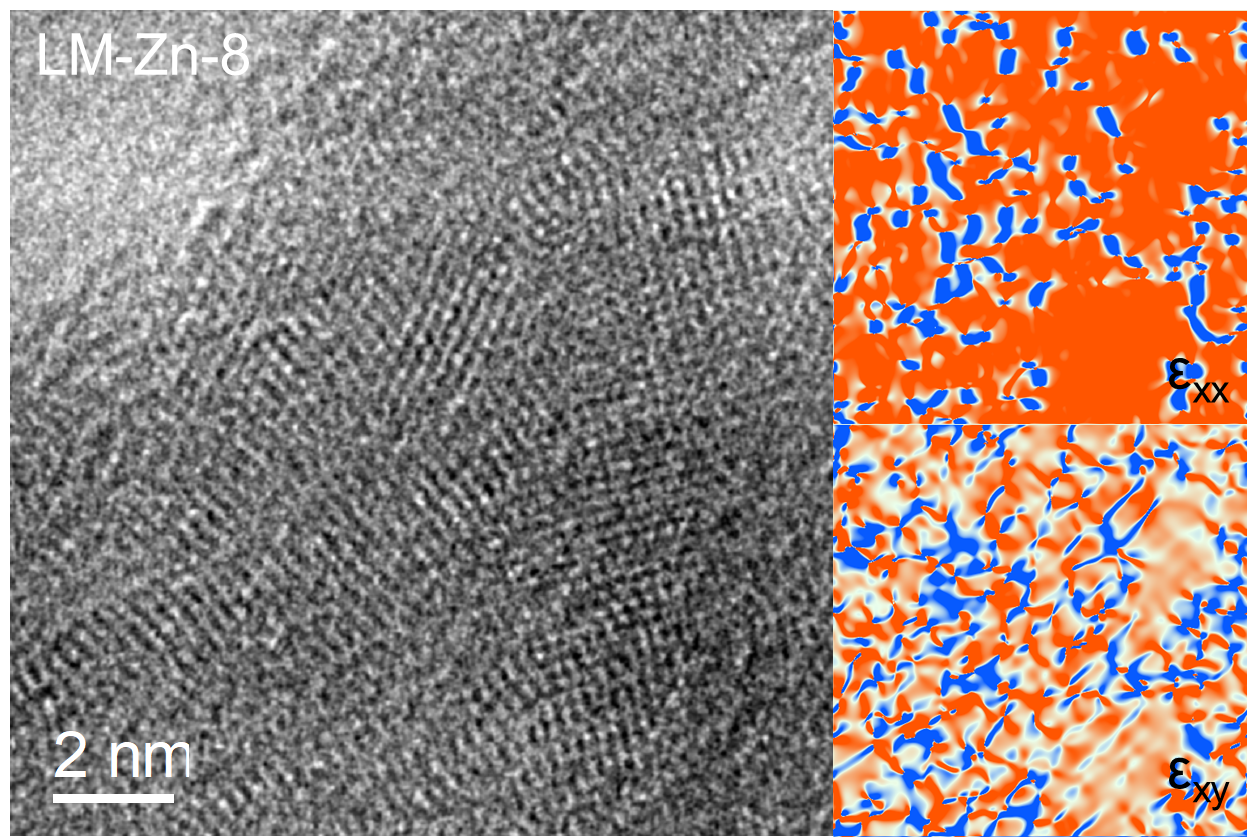


**Figure S16.** The TEM image of LM-Zn-8 and corresponding GPA images.


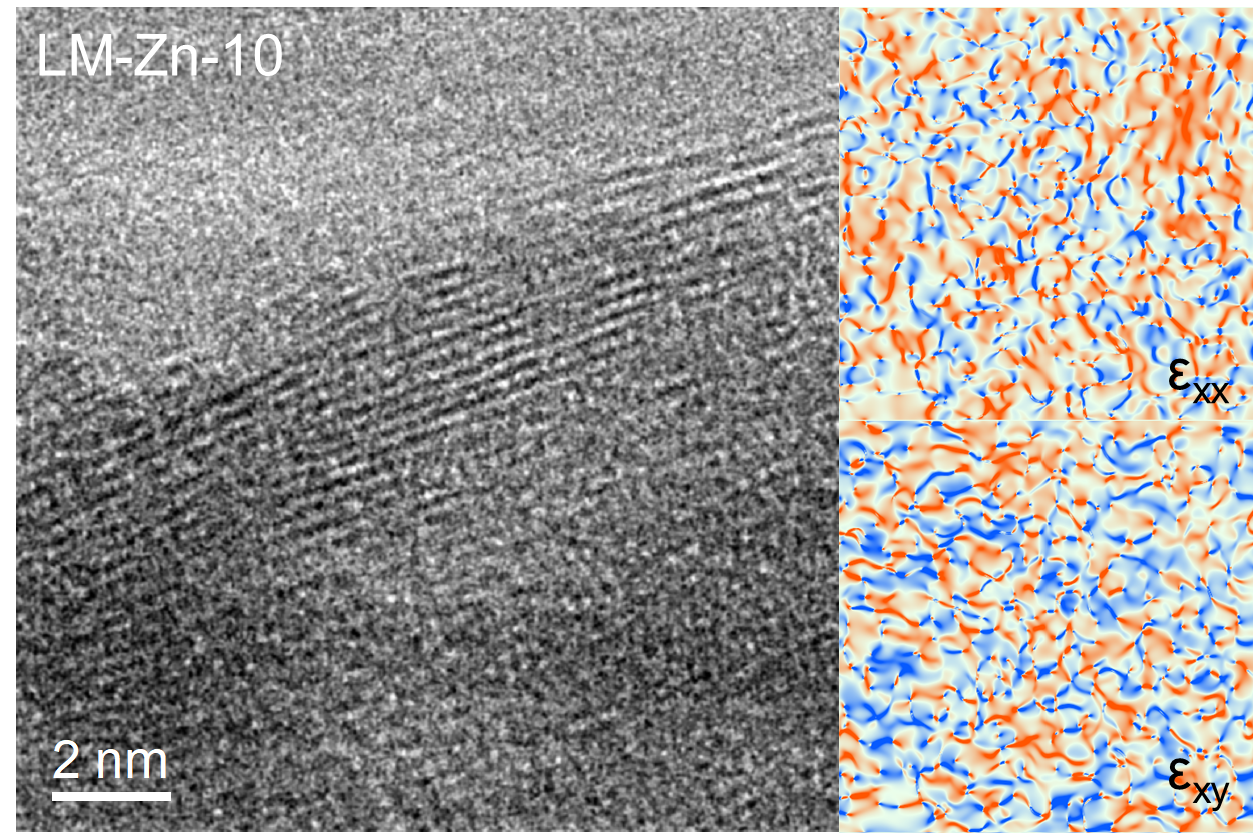


**Figure S17.** The TEM image of LM-Zn-10 and corresponding GPA images.


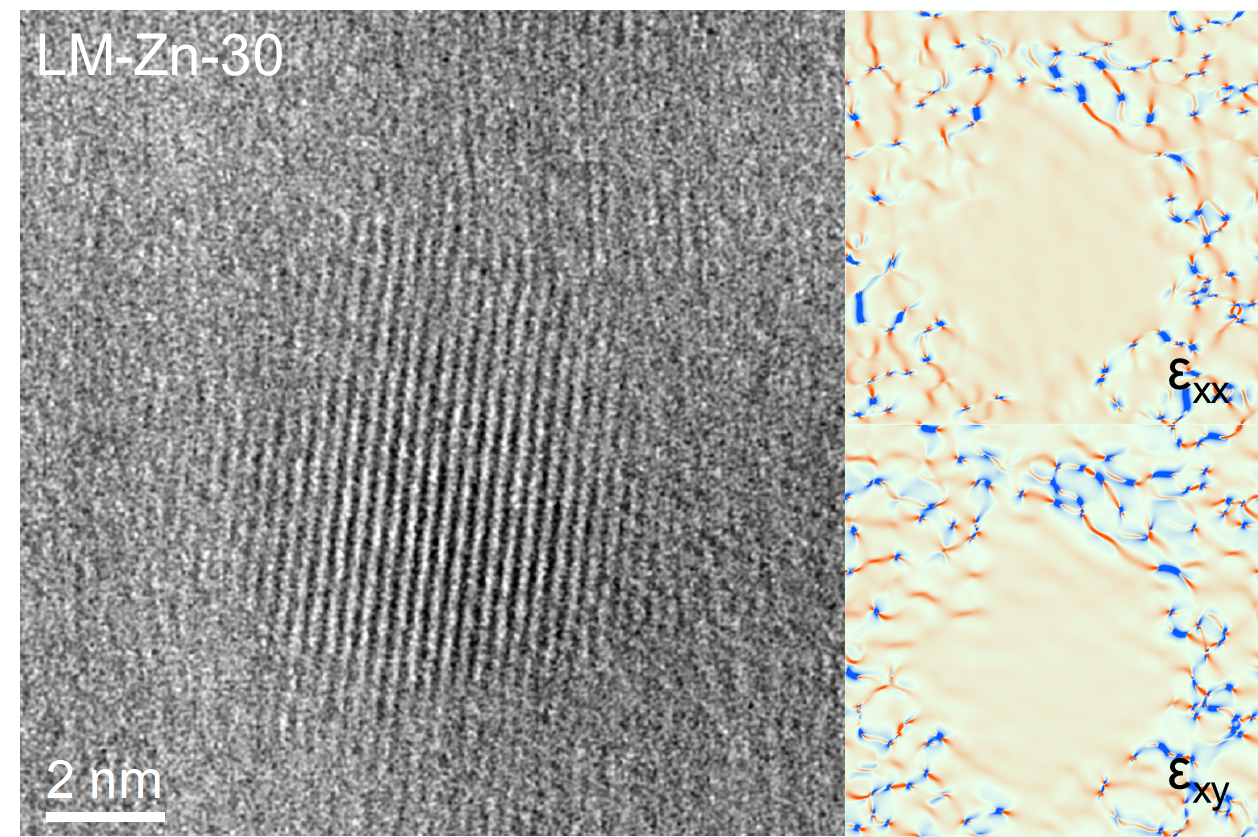


**Figure S18.** The TEM image of LM-Zn-30 and corresponding GPA images.


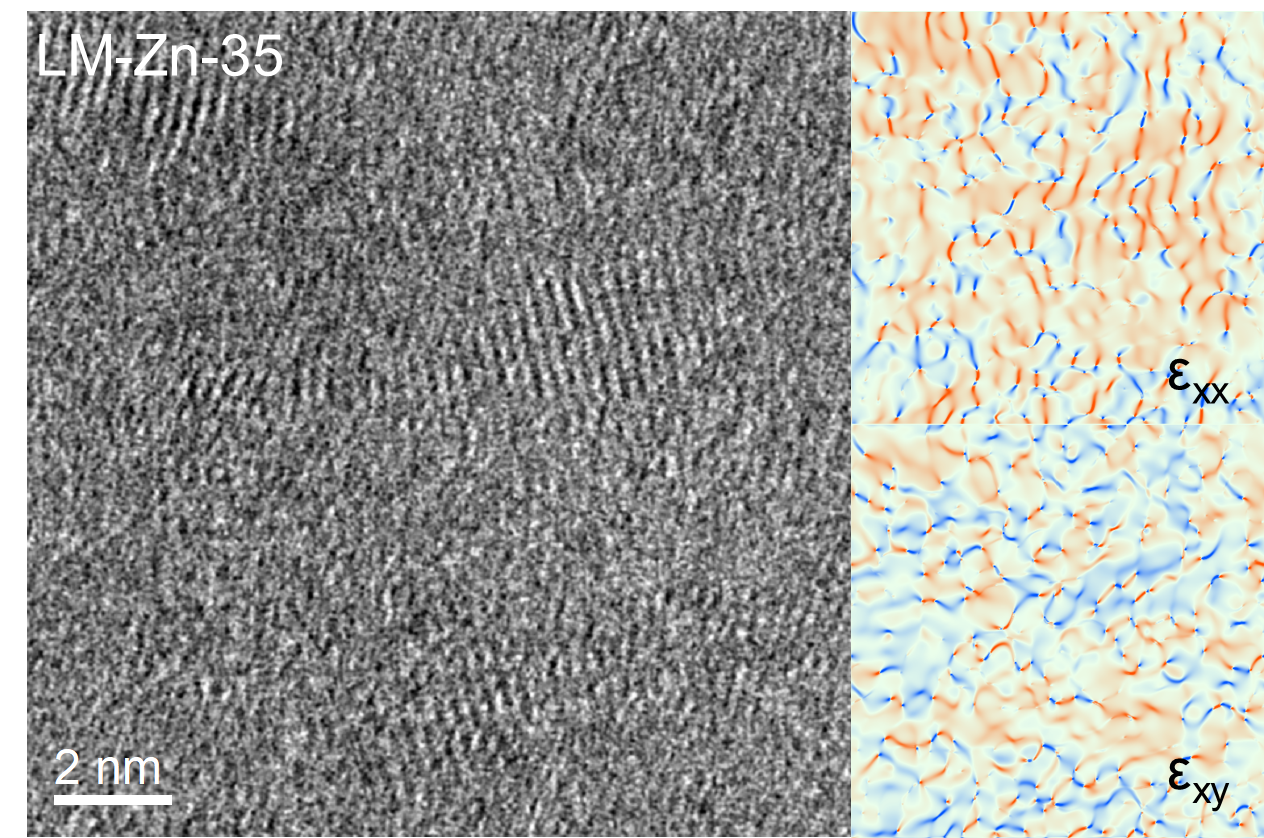


**Figure S19.** The TEM image of LM-Zn-35 and corresponding GPA images.


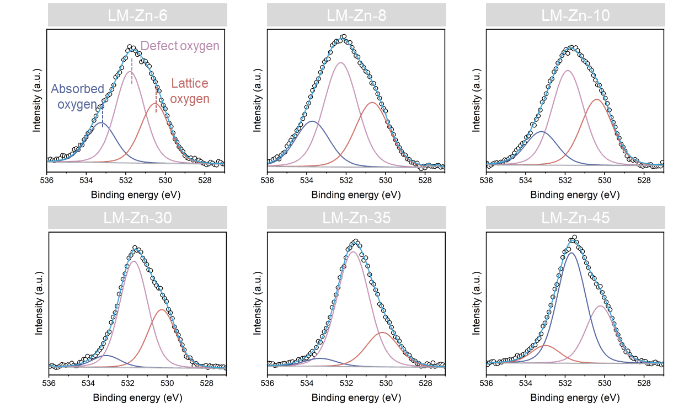


**Figure S20.** The XPS images of O element.


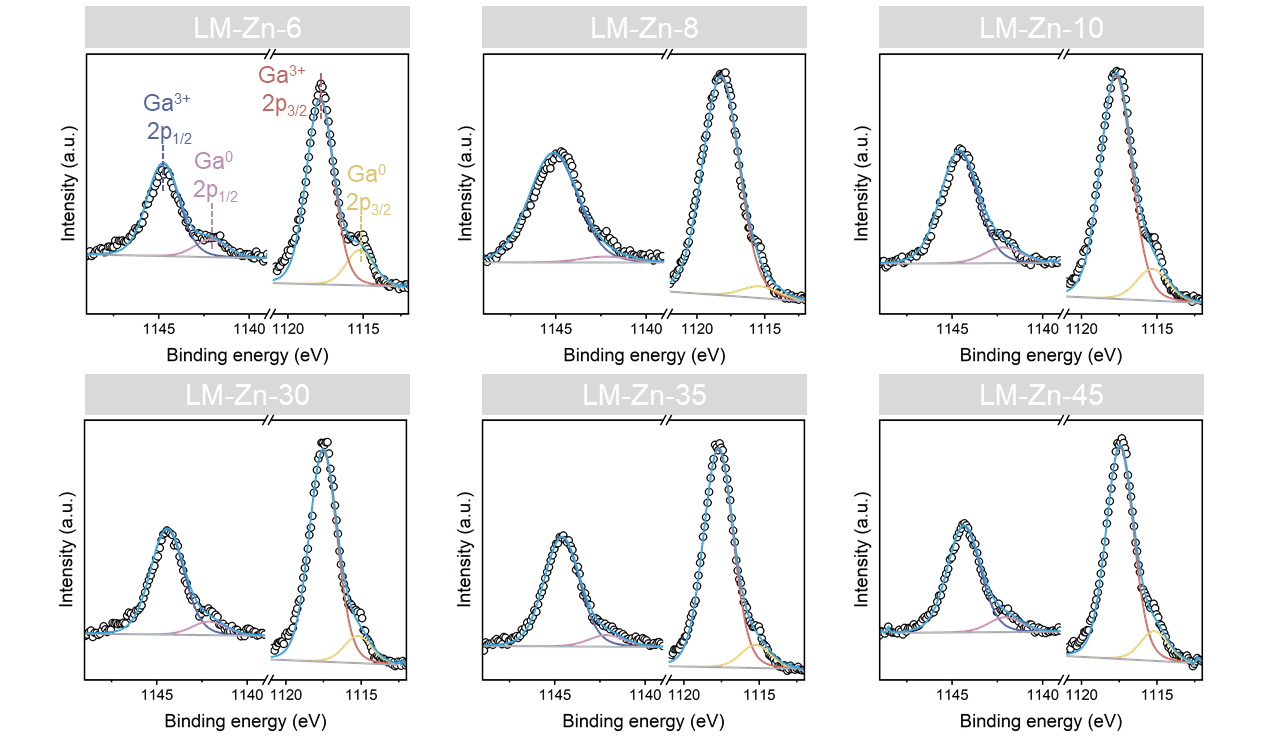


**Figure S21.** The XPS images of Ga element.


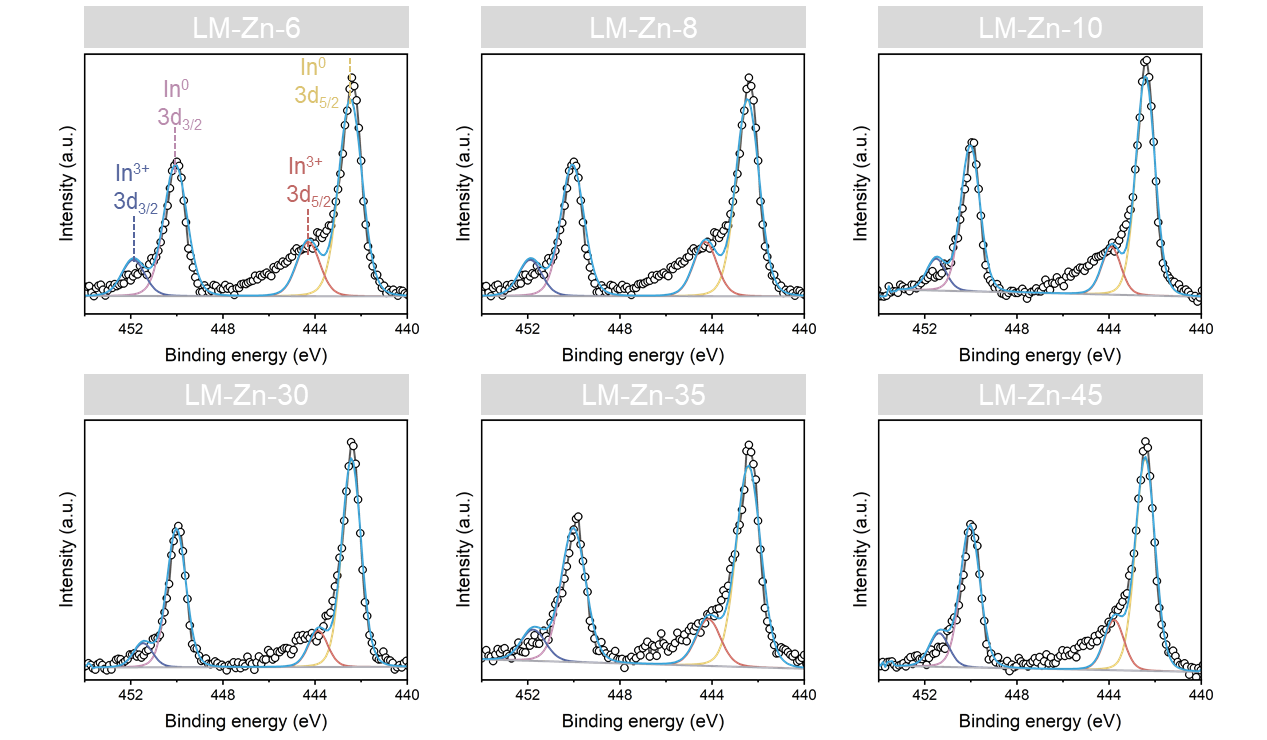


**Figure S22.** The XPS images of In element.


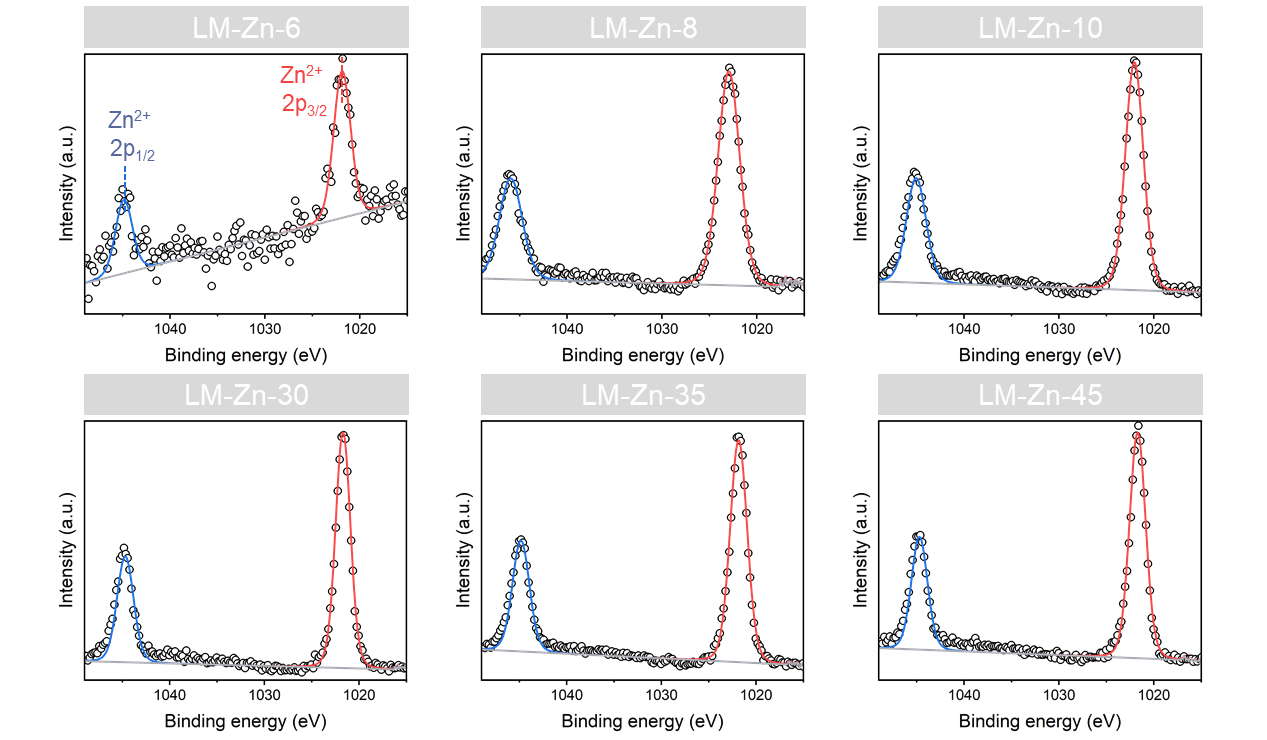


**Figure S23.** The XPS images of Zn element.

The filler loading ratio (70%) was systematically optimized through pre-experimental analysis, balancing dielectric performance and impedance matching: lower ratios (60%) impaired conductive network formation, resulting in insufficient conduction loss and narrow effective absorption bandwidth (EAB), while higher ratios (80%) induced excessive dielectric permittivity (εʹ>20), causing severe impedance mismatch and reflection, as confirmed by electromagnetic parameter simulations (Figure S35, Supporting Information). This ratio also ensured optimal dispersion of LM-Zn particles in the paraffin matrix, avoiding agglomeration, and aligns with established literature ranges (60-70%) for LM-based absorbers to balance loss capability and impedance matching, thus achieving the best comprehensive microwave absorption performance.


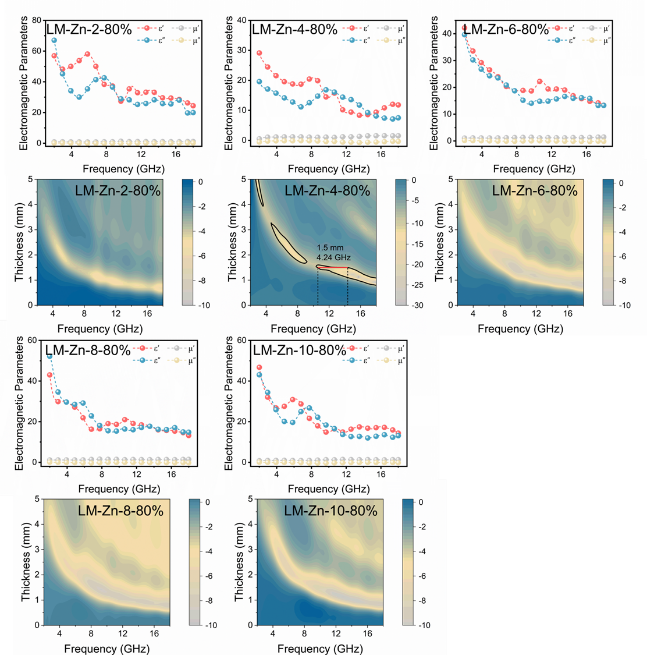


**Figure S24.** Electromagnetic parameters and their calculation of electromagnetic wave absorption performance under filler ration of 80%.


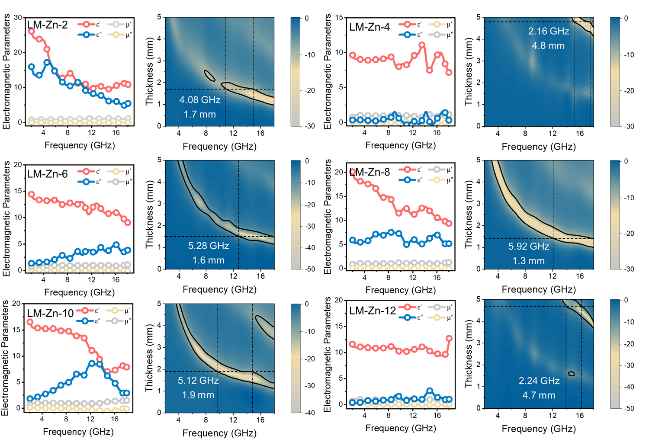


**Figure S25.** Electromagnetic parameters and their calculation of electromagnetic wave absorption performance (LM-Zn-2 to LM-Zn-12).


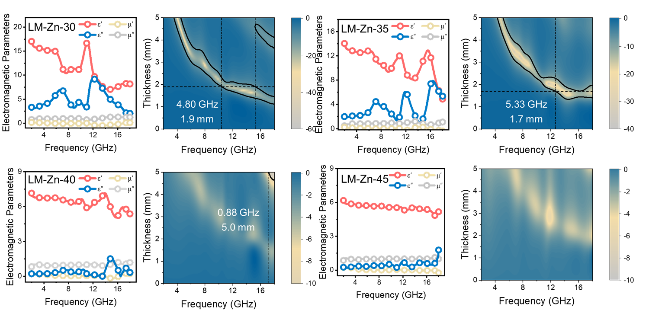


**Figure S26.** Electromagnetic parameters and their calculation of electromagnetic wave absorption performance (LM-Zn-30 to LM-Zn-45).





**Figure S27.** The RL_min_ value of the LM-Zn system at different thicknesses.


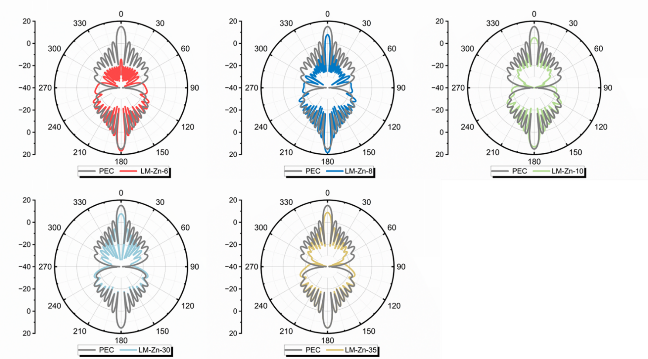


**Figure S28.** The calculated RCS curves of LM-Zn-6, LM-Zn-8, LM-Zn-10, LM-Zn-30 and LM-Zn-35, respectively.


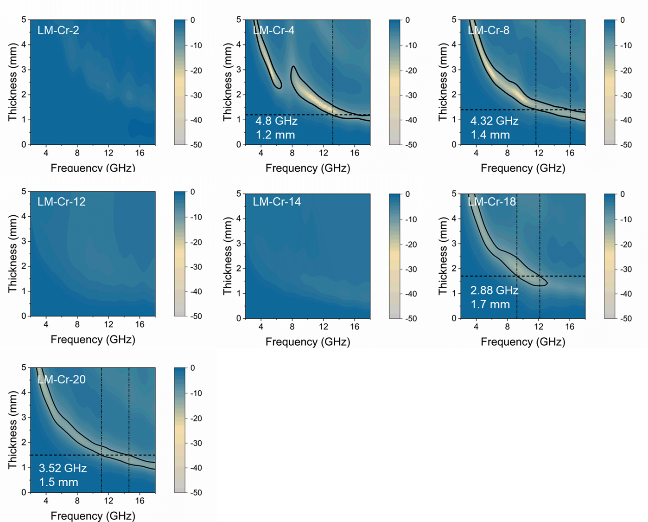


**Figure S29.** The electromagnetic wave absorption performance of LM-Cr.


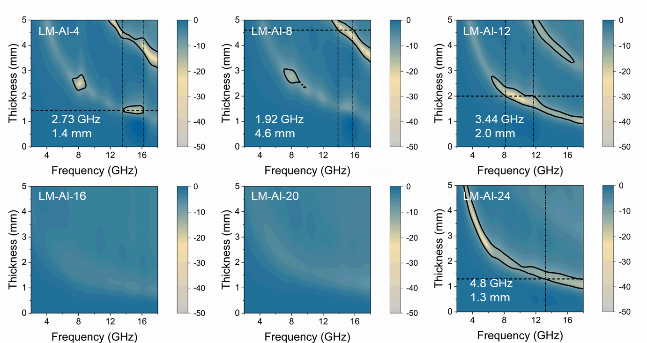


**Figure S30.** The electromagnetic wave absorption performance of LM-Al.


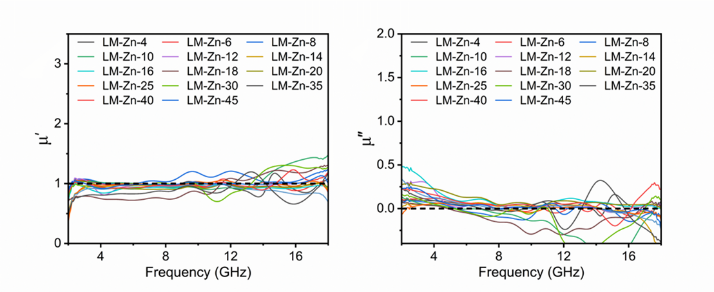


**Figure S31.** The permeability of all samples.


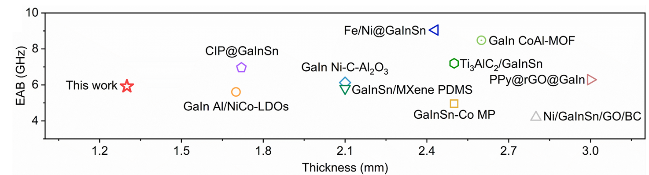


**Figure S32.** The thickness and EAB comparison of LM-Zn-8 with other LM-based EMW absorbing materials.


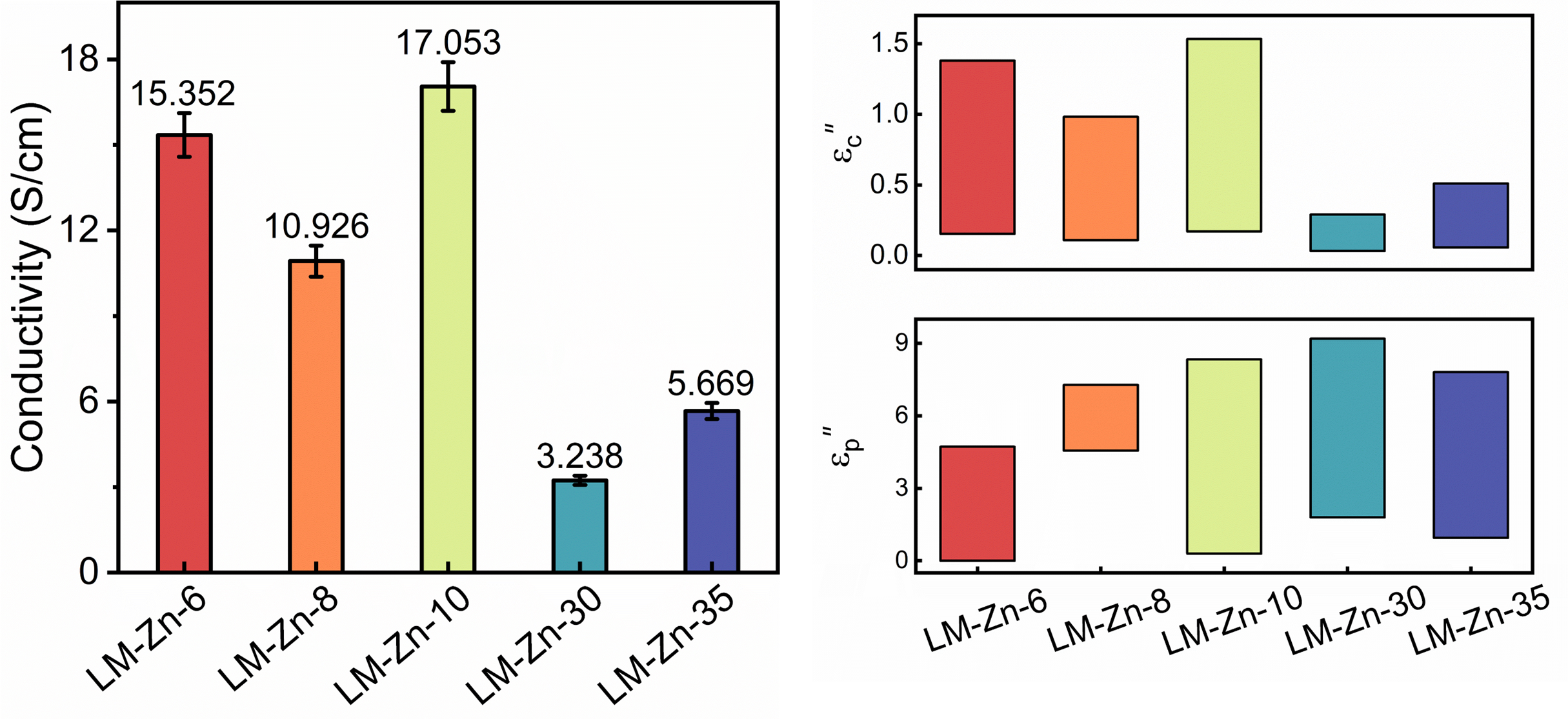


**Figure S33.** The intrinsic conductivity of LM-Zn-6, LM-Zn-8, LM-Zn-10, LM-Zn-30 and LM-Zn-35; the calculated ε_p_" and ε_c_".


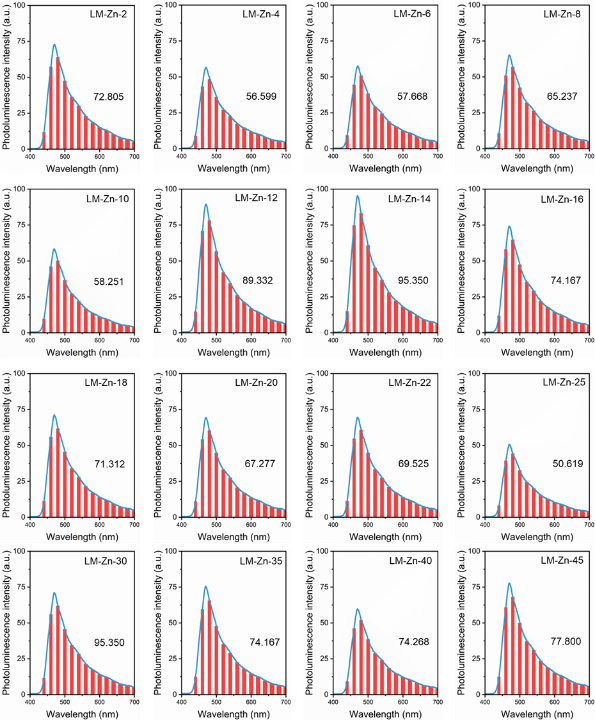


**Figure S34**. Photoluminescence spectrum with an excitation wavelength of 375 nm.


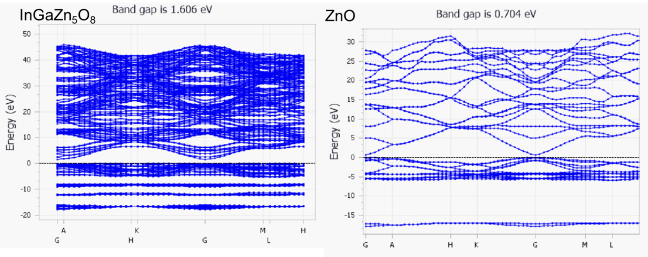


**Figure S35.** The band structures of InGaZn_5_O_8_ and ZnO.


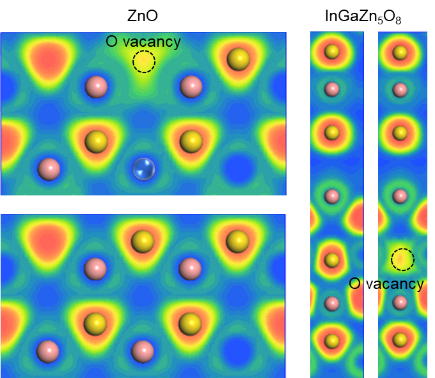


**Figure S36.** The ELF of single oxygen vacancy.

**Table S1.** Samples prepared with different metal ions and different concentrations of metal ions.

| **Sample** | **Metal ions** | **Concentration** |
| --- | --- | --- |
| LM-Zn-4 | Zn | 4 |
| LM-Zn-6 | Zn | 6 |
| LM-Zn-8 | Zn | 8 |
| LM-Zn-10 | Zn | 10 |
| LM-Zn-12 | Zn | 12 |
| LM-Zn-14 | Zn | 14 |
| LM-Zn-16 | Zn | 16 |
| LM-Zn-18 | Zn | 18 |
| LM-Zn-20 | Zn | 20 |
| LM-Zn-22 | Zn | 22 |
| LM-Zn-25 | Zn | 25 |
| LM-Zn-30 | Zn | 30 |
| LM-Zn-35 | Zn | 35 |
| LM-Zn-40 | Zn | 40 |
| LM-Zn-45 | Zn | 45 |
| LM-Al-12 | Al | 12 |
| LM-Al-16 | Al | 16 |
| LM-Al-20 | Al | 20 |
| LM-Al-24 | Al | 24 |
| LM-Cr-2 | Cr | 2 |
| LM-Cr-4 | Cr | 4 |
| LM-Cr-6 | Cr | 6 |
| LM-Cr-8 | Cr | 8 |
| LM-Cr-10 | Cr | 10 |
| LM-Cr-12 | Cr | 12 |
| LM-Cr-14 | Cr | 14 |
| LM-Cr-16 | Cr | 16 |

**Table S2.** High-resolution spectrum, binding energy and peak area of LM-Zn O 1s.

| **Sample** | **Adsorbed O** | | **Area** | **Defect O** | **Area** | **Lattice O** | **Area** |
| --- | --- | --- | --- | --- | --- | --- | --- |
| LM-Zn-6 | 533.2526 | 31.25 | | 531.7912 | 60.75 | 530.5249 | 8.00 |
| LM-Zn-8 | 533.7201 | 21.64 | | 532.2767 | 73.54 | 530.6811 | 4.82 |
| LM-Zn-10 | 533.2090 | 33.12 | | 531.8646 | 60.74 | 530.3982 | 6.14 |
| LM-Zn-30 | 533.0874 | 34.01 | | 531.6943 | 48.82 | 530.2741 | 17.17 |
| LM-Zn-35 | 533.3287 | 30.31 | | 531.6619 | 48.75 | 530.1822 | 20.93 |
| LM-Zn-45 | 533.0017 | 31.28 | | 531.6805 | 48.02 | 530.2162 | 20.07 |

**Table S3.** The proportion of the increase in the effective absorption bandwidth per unit thickness.

| Sample | EAB  (GHz) | Thickness  (mm) | EAB/Thickness  (GHz/mm) | Proportion  (%) |
| --- | --- | --- | --- | --- |
| GaIn Al/NiCo-LDOs | 5.16 | 1.70 | 3.30 | 27.5% |
| GaIn Ni-C-Al_2_O_3_ | 6.13 | 2.10 | 2.92 | 35.8% |
| PPy@rGO@GaIn | 6.29 | 3.00 | 2.10 | 53.8% |
| GaIn CoAl-MOF | 8.48 | 2.60 | 3.26 | 28.4% |
| GaInSn-Co MP | 4.96 | 2.50 | 1.98 | 78.5% |
| Fe/Ni@GaInSn | 9.05 | 2.43 | 3.72 | 18.2% |
| GaInSn/MXene PDMS | 5.80 | 2.10 | 2.76 | 39.3% |
| Ti_3_AlC_2_/GaInSn | 7.20 | 2.50 | 2.88 | 27.5% |
| This work | 5.92 | 1.30 | 4.55 |  |

**Table S4.** The thickness and EAB comparison of LM-Zn-8 with other EMW absorbing materials.

| Sample | EAB  (GHz) | Thickness  (mm) | EAB/Thickness  (GHz/mm) | Ref. |
| --- | --- | --- | --- | --- |
| Cu_3_Se_2_/rGO | 8.24 | 2.20 | 3.75 | [1] |
| MXene/Fe_3_O_4_/PDMS | 4.20 | 2.05 | 2.05 | [44] |
| ZIF-8@ZIF-67 | 6.00 | 2.67 | 2.25 | [42] |
| Dy_2_O_3_@SWCNT | 7.80 | 2.80 | 2.79 | [45] |
| N-CNF@MXene@MoS_2_ | 7.58 | 2.50 | 3.03 | [43] |
| Cu/Fe@NC | 5.09 | 1.62 | 3.14 | [46] |
| CoFe_2_O_4_ NF@N-rGO | 8.70 | 3.00 | 2.9 | [47] |
| This work | 5.92 | 1.30 | 4.55 |  |

**Reference**

[S1] Z. H. Zhao, Y. C. Qing, L. Kong, H. L. Xu, X. M. Fan, J. J. Yun, L. M. Zhang, H. J. Wu, *Adv. Mater.* **2023**, 36, 2304182.

[S2] J. Q. Tao, J. T. Zhou, Z. J. Yao, J. Wang, L. L. Xu, X. W. Tao, H. J. Wu, *Adv. Funct. Mater.* **2024**, 34, 2310640.

[S3] W. B. Deng, T. H Li, H. Li, J. abdul, L. T. Liu, A. Dang, X. Liu, M. F. Duan, H. J. Wu, *Small* **2024**, 20, 2309806.
